# Candidate genes on murine chromosome 8 are associated with susceptibility to *Staphylococcus aureus* infection in mice and are involved with *Staphylococcus aureus* septicemia in humans

**DOI:** 10.1371/journal.pone.0179033

**Published:** 2017-06-08

**Authors:** Qin Yan, Sun Hee Ahn, Felix Mba Medie, Batu K. Sharma-Kuinkel, Lawrence P. Park, William K. Scott, Hitesh Deshmukh, Ephraim L. Tsalik, Derek D. Cyr, Christopher W. Woods, Chen-Hsin Albert Yu, Carlton Adams, Robert Qi, Brenda Hansen, Vance G. Fowler

**Affiliations:** 1 Division of Infectious Diseases & International Health, Department of Medicine, Duke University School of Medicine, Durham, North Carolina, United States of America; 2 Department of Biochemistry School of Dentistry, Chonnam National University, Bukgu, Gwangju, Korea; 3 Duke Global Health Institute, Duke University, Durham, North Carolina, United States of America; 4 Dr. John T. Macdonald Foundation Department of Human Genetics and John P. Hussman Institute for Human Genomics, University of Miami, Miami, Florida, United States of America; 5 Department of Pediatrics, University of Cincinnati, Cincinnati, Ohio, United States of America; 6 Emergency Medicine Service, Durham Veteran’s Affairs Medical Center, Durham, North Carolina, United States of America; 7 Duke Clinical Research Institute, Durham, North Carolina, United States of America; 8 Section on Infectious Diseases, Durham Veteran’s Affairs Medical Center, Durham, North Carolina, United States of America; University of Mississippi Medical Center, UNITED STATES

## Abstract

We previously showed that chromosome 8 of A/J mice was associated with susceptibility to *S*. *aureus* infection. However, the specific genes responsible for this susceptibility are unknown. Chromosome substitution strain 8 (CSS8) mice, which have chromosome 8 from A/J but an otherwise C57BL/6J genome, were used to identify the genetic determinants of susceptibility to *S*. *aureus* on chromosome 8. Quantitative trait loci (QTL) mapping of *S*. *aureus*-infected N2 backcross mice (F1 [C8A] × C57BL/6J) identified a locus 83180780–88103009 (GRCm38/mm10) on A/J chromosome 8 that was linked to *S*. *aureus* susceptibility. All genes on the QTL (n~ 102) were further analyzed by three different strategies: 1) different expression in susceptible (A/J) and resistant (C57BL/6J) mice only in response to *S*. *aureus*, 2) consistently different expression in both uninfected and infected states between the two strains, and 3) damaging non-synonymous SNPs in either strain. Eleven candidate genes from the QTL region were significantly differently expressed in patients with *S*. *aureus* infection vs healthy human subjects. Four of these 11 genes also exhibited significantly different expression in *S*. *aureus*-challenged human neutrophils: *Ier2*, *Crif1*, *Cd97* and *Lyl1*. CD97 ligand binding was evaluated within peritoneal neutrophils from A/J and C57BL/6J. CD97 from A/J had stronger CD55 but weaker integrin α5β1 ligand binding as compared with C57BL/6J. Because CD55/CD97 binding regulates immune cell activation and cytokine production, and integrin α5β1 is a membrane receptor for fibronectin, which is also bound by *S*. *aureus*, strain-specific differences could contribute to susceptibility to *S*. *aureus*. Down-regulation of *Crif1* with siRNA was associated with increased host cell apoptosis among both naïve and *S*. *aureus*-infected bone marrow-derived macrophages. Specific genes in A/J chromosome 8, including *Cd97* and *Crif1*, may play important roles in host defense against *S*. *aureus*.

## Introduction

An emerging body of evidence supports the concept that human genetic variation can influence host susceptibility to and outcome of infectious diseases. Examples of human genetic variation and susceptibility to specific infectious syndromes include susceptibility to severe sepsis in Chinese Han subjects with the rs1800629 variant of the TNF gene [1], genetic variants of TRAF6 and increased susceptibility to sepsis-induced acute lung injury [2], variants in β2-adrenocepter and an increased susceptibility to bacterial meningitis [3], Toll-like receptor variants associated with both infectious and autoimmune diseases [4], and IL17A variation in association with susceptibility to Gram-positive infection and severe sepsis [5].

However, the genetic basis for variation in host susceptibility to *S*. *aureus* remains largely unknown. We [6–8] and others [9, 10] have reported the different susceptibility to *S*. *aureus* in various inbred mouse strains. For example, A/J is highly susceptible to *S*. *aureus* infection while C57BL/6J is resistant [9]. These two strains thus provide a unique platform to investigate the host genetic determinants associated with susceptibility to *S*. *aureus* infection. Using these strains, we previously reported that the genetic determinants of susceptibility to *S*. *aureus* in A/J mice localized to chromosomes 8, 11, and 18 [6, 8] and identified candidate susceptibility genes on chromosomes 11 [8] and 18 [6].

In the present investigation, we used a multipronged strategy to identify genes associated with susceptibility to *S*. *aureus* infection on murine chromosome 8. We initially localized the region on chromosome 8 associated with *S*. *aureus* susceptibility by quantitative trait locus (QTL) mapping. Having narrowed down our investigation to this region of ~ 100 genes, we employed a comprehensive approach to address multiple potential mechanisms by which genetic variation could result in our phenotype of interest. First, we considered the possibility that genetic susceptibility to *S*. *aureus* was due to genes that were only differentially expressed between A/J and C57BL/6J in the setting of active *S*. *aureus* infection. Second, we considered the possibility that susceptibility to *S*. *aureus* infection was due to genes that were differentially expressed between susceptible A/J and resistant C57BL/6J mice in both uninfected and *S*. *aureus* infected states. In our third approach, we considered the possibility that damaging single nucleotide polymorphisms (SNPs) in key genes influenced susceptibility to *S*. *aureus* infection. We then used whole blood gene expression data from a cohort of patients with *S*. *aureus* blood stream infection (BSI) to identify relevance of the candidate genes in human infection [11]. Finally, we evaluated the biological plausibility of our top two priority genes, *Cd97* and *Crif1*, as important determinants of susceptibility to *S*. *aureus* infection.

## Results

### QTL mapping identifies locus on chromosome 8 linked to susceptibility to *S*. *aureus*

**Fig 1:**
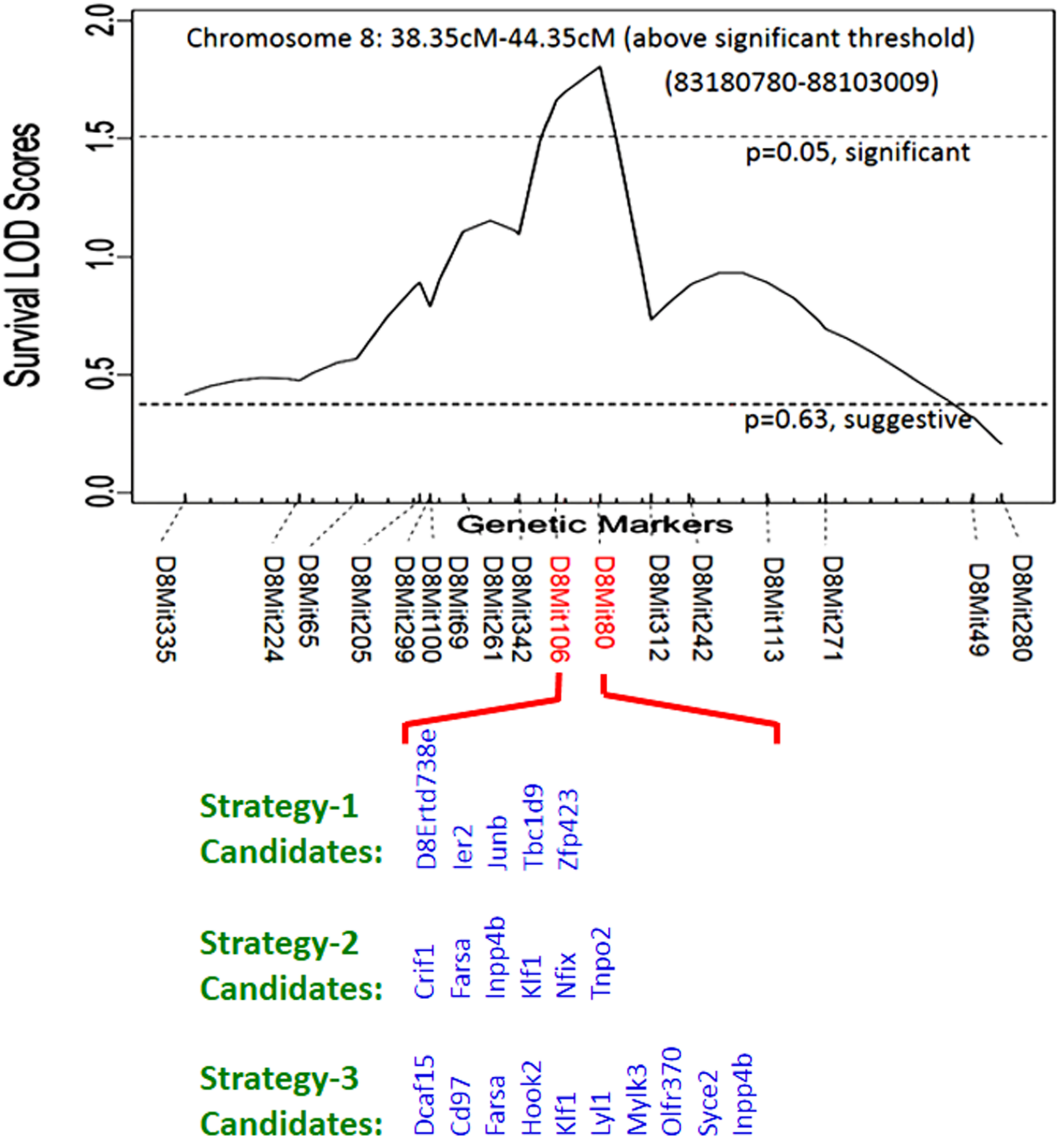
Chromosome substitution strain 8 (CSS8) mice were susceptible to *S*. *aureus* infection and QTL mapping found a region with putative candidate genes on chromosome 8. Chromosome 8 LOD score plot for susceptibility to *S*. *aureus* in N2 backcross mice (F1 [C8A] × C57BL/6J). A total of 337 intercross mice (both sexes; age 6 to 8 weeks) were injected via intraperitoneal route with 10^7^ CFU/g *S*. *aureus* Sanger 476 and observed every 8 hours continuously for 5 days. Thresholds for significant (p = 0.05) and suggestive (p = 0.63) linkage are indicated by the horizontal dashed lines. LOD score was determined by the J/qtl permutation test using 1,000 permuted data sets. The microsatellite markers for determining genotypes of N2 backcross mice are marked along the X-axis.

Previously we demonstrated that C57BL/6J inbred mice were resistant to *S*. *aureus* sepsis (median survival >5 days) [6]. By contrast, A/J and Chromosomal Substitution Strain 8 (CSS8) mice, which contain A/J chromosome 8 but are otherwise genetically C57BL/6J, were susceptible to *S*. *aureus* challenge, with significantly lower median survival (<2 days) and significantly higher kidney bacterial load 24 hours following *S*. *aureus* challenge[6]. The A/J derived allele on chromosome 8 that is responsible for *S*. *aureus* susceptibility is dominant, and F1 mice of CSS8 × C57BL/6J were susceptible to *S*. *aureus* [6]. Mouse gender did not influence *S*. *aureus* susceptibility [6]. In the current manuscript, we employed QTL mapping to localize genetic regions on chromosome 8 that are associated with susceptibility to *S*. *aureus*. Using 337 *S*. *aureus*-infected N2 (F1 [C8A] × C57BL/6J) backcross mice, QTL mapping identified a region on chromosome 8 that was significantly linked to survival time after *S*. *aureus* infection. This region was located between 83180780–88103009 (GRCm38/mm10) and contained approximately 102 genes (Fig 1). A/J and CSS8 also demonstrate similar patterns of susceptibility to infection with *Escherichia coli* (S1 Fig).

**Fig 2:**
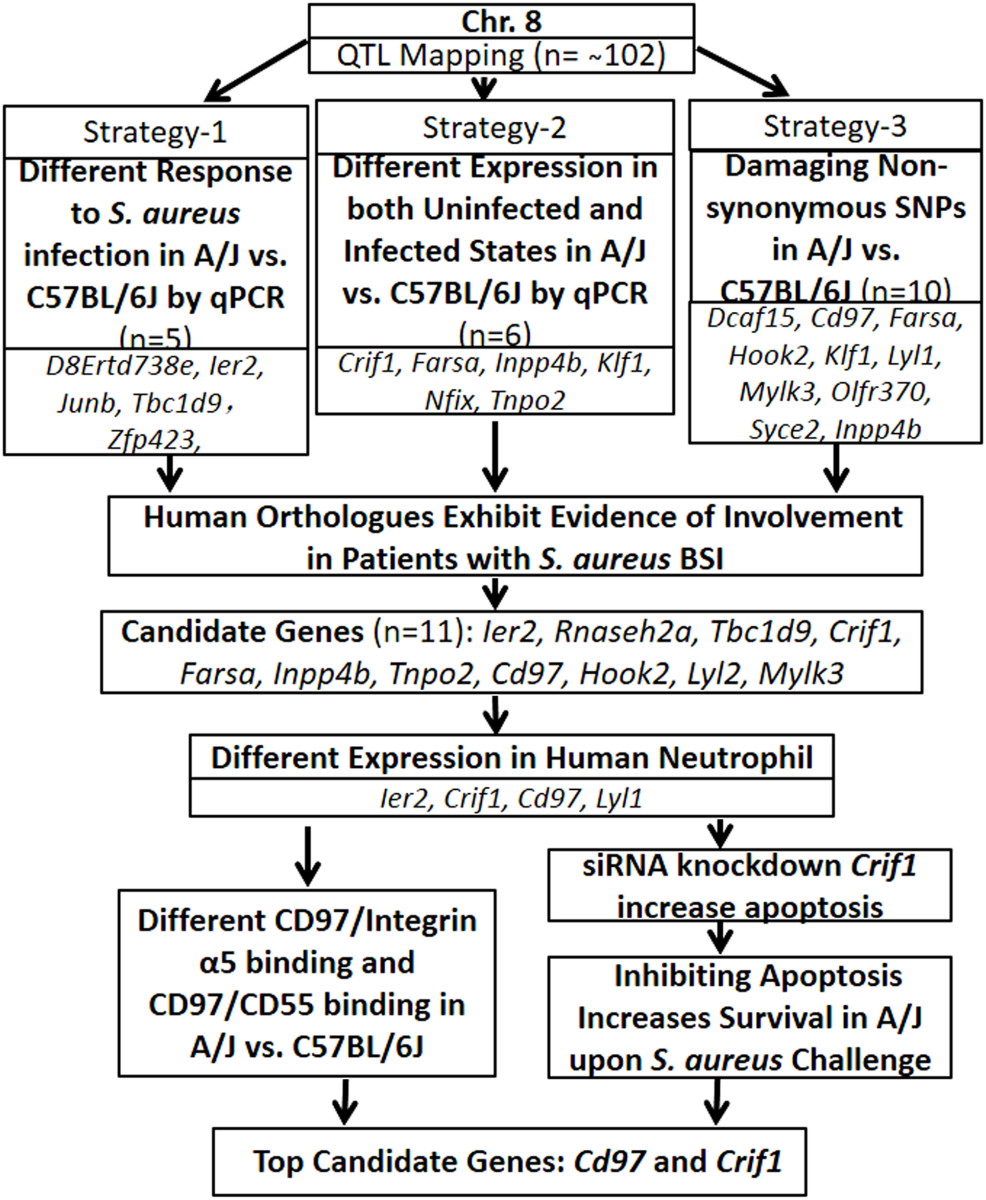
Overall strategies for identifying genes associated with *S*. *aureus* susceptibility on chromosome 8 of A/J mice. Flow chart of the strategies for identifying *S*. *aureus* susceptible genes on chromosome 8 of A/J mice. Three different strategies were applied. *Strategy 1* identified genes within the QTL region that were differentially expressed between A/J and C57BL/6J mice only in the setting of *S*. *aureus* infection by microarray and qPCR. Five candidates were identified by Strategy 1: *D8Ertd738e*, *Ier2*, *Junb*, *Tbc1d9* and *Zfp423*. *Strategy 2* identified genes within the QTL region that were differentially expressed between A/J and C57BL/6J mice in both uninfected and *S*. *aureus* infected states by microarray and qPCR. Six candidates were identified by Strategy 2: *Crif1*, *Farsa*, *Inpp4b*, *Klf1*, *Nfix* and *Tnpo2*. *Strategy 3* identified damaging non-synonymous SNPs in A/J vs. C57BL/6J. Ten candidates were identified by Strategy 3: *Dcaf15*, *Cd97*, *Farsa*, *Hook2*, *Klf1*, *Lyl1*, *Mylk3*, *Olfr370*, *Syce2* and *Inpp4b*.

Next, we sought to localize the basis of susceptibility to *S*. *aureus* within the QTL region by testing three possible sources of genetic variation: 1) genes within the QTL that are differentially expressed between susceptible A/J and resistant C57BL/6J mice only in *S*. *aureus* infected state; 2) genes within the QTL that are differentially expressed between susceptible A/J and resistant C57BL/6J mice in both uninfected and *S*. *aureus* infected states; and 3) presence of damaging SNPs in genes within the QTL that result in a damaged or dysfunctional gene product (Fig 2).

### Identification of candidate genes within the QTL region

#### Strategy 1: Five genes within the identified QTL are significantly differentially expressed between A/J and C57BL/6J only in *S*. *aureus* infection and are validated by qPCR

**Fig 3:**
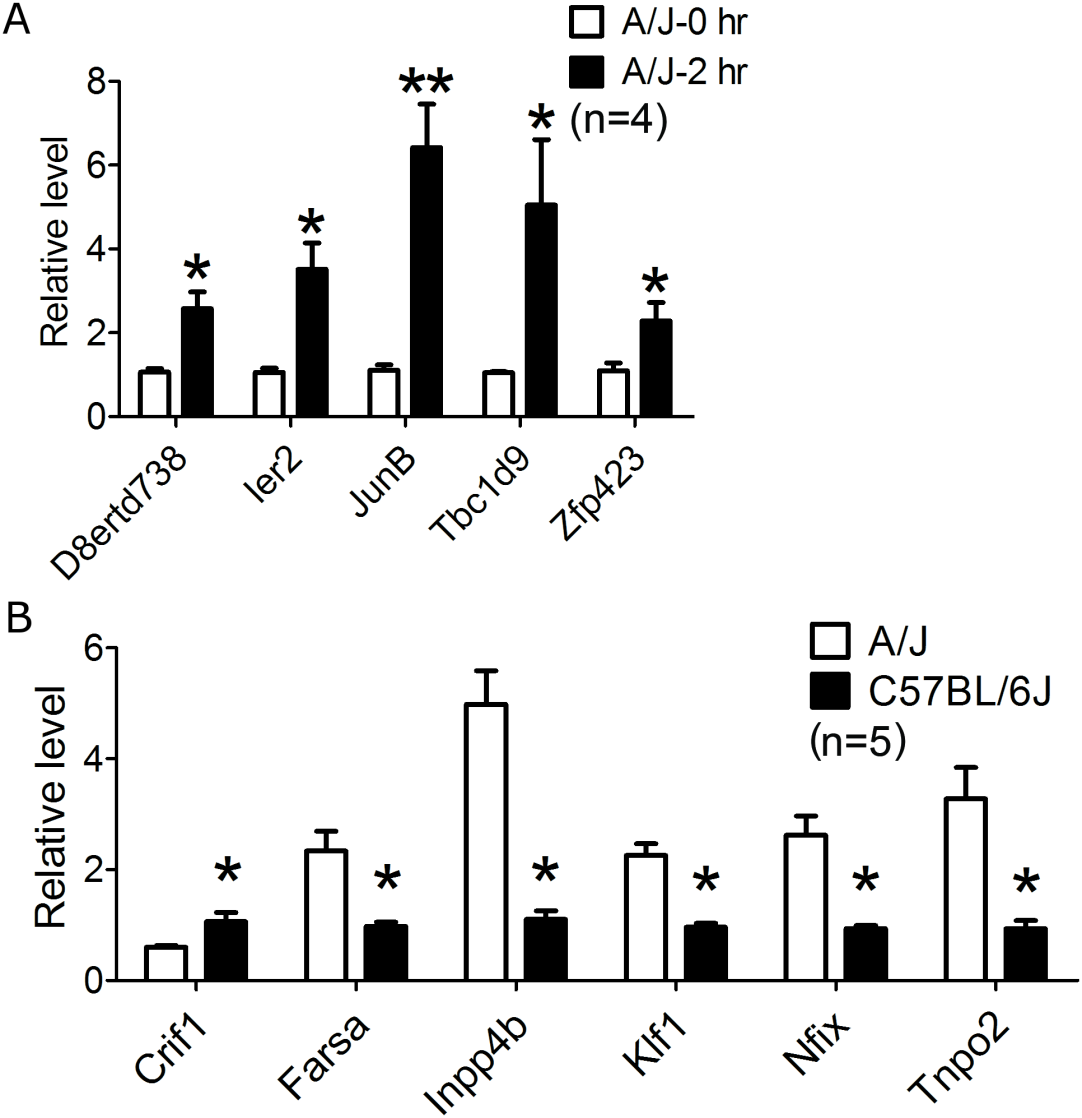
qPCR validation of murine candidate genes. **(A) qPCR validation of genes identified by Strategy 1.** Genes within the QTL region that were differentially expressed between A/J and C57BL/6J mice only in the setting of *S*. *aureus* infection by microarray (S1 Table) underwent qPCR validation. qPCR validated five genes (*D8ertd738*, *Ier2*, *JunB*, *Tbc1d9*, *Zfp423*) with expression patterns in A/J and C57BL/6J (n = 4 in each group) that were consistent with microarray results. At 2 hours post *S*. *aureus* infection, the fold difference of A/J-2hr vs A/J-0hr was *D8ertd738* (2.58fold, p<0.05), *Ier2* (3.51 fold, p<0.05), *JunB* (6.42 fold, p<0.01), *Tbc1d9* (7.05 fold, p<0.05) *Zfp423* (2.29 fold, p<0.05). “*” represents p<0.05, “**” represents p<0.01. **(B) qPCR validation of genes identified by Strategy 2.** Genes within the QTL region that were differentially expressed between A/J and C57BL/6J mice in both uninfected and *S*. *aureus* infected states by microarray (S2 Table) underwent qPCR validation. qPCR validated six genes (*Crif1*, *Farsa*, *Inpp4b*, *Klf1*, *Nfix*, and *Tnpo2*) with expression patterns in A/J and C57BL/6J (n = 5 in each group) that were consistent with microarray results. At 3 hours post *S*. *aureus* infection, the fold difference of A/J vs C57BL/6J was *Crif1* (0.6fold, p<0.05), *Farsa* (2.4fold, p<0.05), *Inpp4b* (4.5fold,p<0.05), *Klf1* (2.4fold, p<0.05) *Nfix* (2.8fold, p<0.05), and *Tnpo2* (3.5fold, p<0.05). “*” represents p<0.05. All mice were 8-week old males.

First, we considered the possibililty that candidate genes within the QTL would exhibit similar expression levels between uninfected susceptible and resistant inbred strains but would be differentially expressed in the setting of *S*. *aureus* infection. After adjustment for multiple comparisons in microarray results, 8 genes, all in A/J, exhibited similar expression levels between uninfected susceptible and resistant inbred strains but were significantly differentially expressed between pre-infection state and at 2 hours following *S*. *aureus* infection (S1 Table). Of these genes, 5 were validated by qPCR: *D8ertd738*; *Ier2*; *JunB*; *Tbc1d9; Zfp423*. These 5 genes exhibited significantly different qPCR-measured expression in A/J at 2hr following *S*. *aureus* infection vs uninfected A/J: *D8ertd738* (2.58fold, p<0.05); *Ier2* (3.51 fold, p<0.05); *JunB* (6.42 fold, p<0.01); *Tbc1d9* (7.05 fold, p<0.05); and *Zfp423* (2.29 fold, p<0.05) (Fig 3A). These 5 genes comprised our initial candidate gene list for Strategy 1 (Fig 2).

#### Strategy 2: Six genes within the QTL region are significantly differentially expressed between A/J and C57BL/6J at all pre-infection and post-*S*. *aureus* infection timepoints and are validated by qPCR

A total of 12 genes within the identified QTL region were significantly differentially expressed between susceptible A/J and resistant C57BL/6J at all pre-infection and post-infection time points from microarray (S2 Table). Of these, 6 were validated by qPCR: *Crif1*, *Farsa*, *Inpp4b*, *Klf1*, *Nfix*, and *Tnpo2* (Fig 3B). Using qPCR at 3hr post *S*. *aureus* infection, *Crif1* expression was significantly lower in A/J as compared with C57BL/6J (0.6fold; p<0.05), while other 5 genes exhibited significantly higher expression in A/J (*Farsa* [2.4fold, p<0.05], *Inpp4b* [4.5fold, p<0.05], *Klf1* [2.4fold, p<0.05], *Nfix* [2.8fold, p<0.05], and *Tnpo2* [3.5fold, p<0.05]). These 5 genes comprised our candidate gene list for Strategy 2 (Fig 2).

#### Strategy 3: Identifying damaging SNPs within the QTL region of susceptible A/J or resistant C57BL/6J mice

To consider the possibility that the genetic basis for susceptibility to *S*. *aureus* might be the presence of damaging SNPs, we performed S.I.F.T. analysis on all known non-synonymous mutations for genes within the QTL region on murine chromosome 8. A total of 10 genes within the QTL region were predicted to contain damaging non-synonymous sequence variants in the A/J or C57BL/6NJ mouse strains: *Dcaf15*, *Cd97*, *Farsa*, *Hook2*, *Klf1*, *Lyl1*, *Mylk3*, *Olfr370*, *Syce2*, *Inpp4b* (Fig 2). Damaging SNPs were identified in A/J in 7 of the 10 genes (*Dcaf15*, *Farsa*, *Hook2*, *Lyl1*, *Mylk3*, *Olfr370*, *Syce2*) and in C57BL/6J in 5 of the 10 genes (*Cd97*, *Hook2*, *Klf1*, *Mylk3*, *Inpp4b*). These 10 genes comprised our candidate gene list for Strategy 3 (Fig 2).

#### Evidence for involvement of candidate genes in human *S*. *aureus* infection

**Fig 4:**
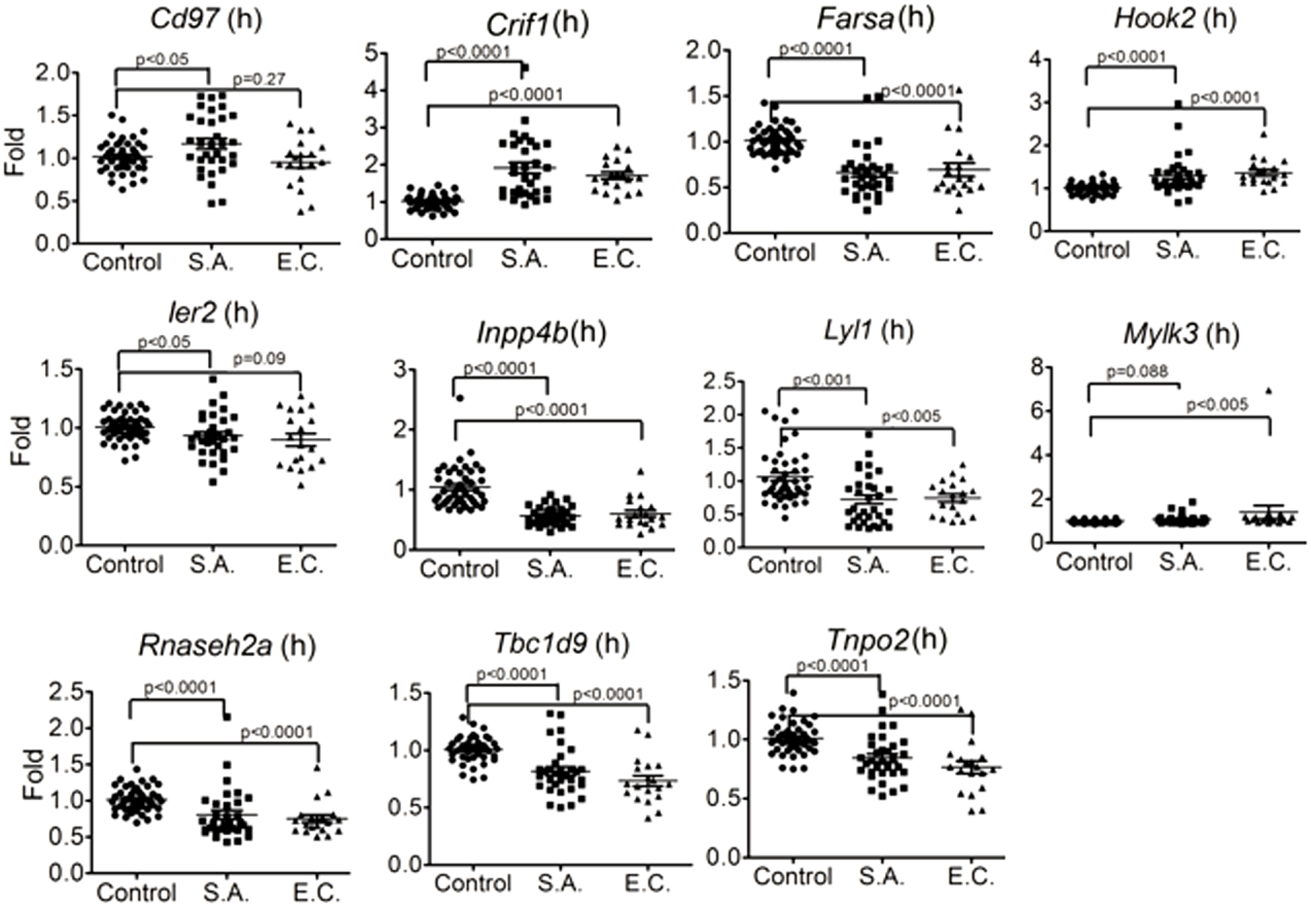
Human orthologues of 11 candidate genes were significantly differentially expressed between patients with blood stream infection (BSI) due to *S*. *aureus* (S. A.), *E*. *coli* (E.C.) and healthy subjects (Control). Human orthologues of 11 candidate genes (*Crif1*, *Farsa*, *Inpp4b*, *Tnpo2*, *Cd97*, *Hook2*, *Ier2*, *Lyl1*, *Mylk3*, *Rnaseh2a* and *Tbc1d9*) were significantly differentially expressed between patients with *S*. *aureus* BSI and healthy subjects by microarray. Human blood RNA from patients with *S*. *aureus* BSI (n = 32) and healthy subjects with no infection (n = 44) were extracted and analyzed and applied to microarray. The expression of *Cd97* (1.17 fold; p<0.05), *Crif1* (1.87 fold; p<0.0001), *Hook2* (1.30 fold; p = 0.0001) were significantly higher in *S*. *aureus* BSI patients as compared with healthy controls. By contrast, the expression of *Farsa* (0.66fold; p<0.0001), *Ier2* (0.93 fold; p<0.05), *Inpp4b* (0.54fold; p<0.0001), *Lyl1* (0.72 fold; p<0.001), *Rnaseh2a* (0.81 fold; p = 0.001), *Tbc1d9* (0.51 fold; p<0.0001) and *Tnpo2* (0.84fold; p<0.0001), were significantly lower in *S*. *aureus* BSI patients. All of the 11 genes except *Cd97* showed similar expression changes in *Escherichia coli* BSI (n = 19) patients.

To provide evidence for the relevance of our findings in human infections, we evaluated whether human orthologues of the murine candidate genes identified by the three approaches were differentially expressed in patients with *S*. *aureus* BSI as compared to healthy human controls using gene expression data. The demographic and clinical details of these patients have been previously published (https://doi.org/10.1371/journal.pone.0048979.t001) [11]. Eleven of the putative candidate genes had human orthologues that were significantly differentially expressed between patients with *S*. *aureus* BSI (n = 32) and healthy subjects (n = 44) (*Ier2*, *Rnaseh2a*, *Tbc1d9*, *Crif1*, *Farsa*, *Inpp4b*, *Tnpo2*, *Cd97*, *Hook2*, *Lyl2*, *Mylk3)* (Fig 4). Three genes had increased levels of expression in *S*. *aureus* BSI patients: *Cd97* (1.16-fold, p<0.05), *Crif1*(1.87-fold; p<0.0001), *and Hook2* (1.29-fold, p = 0.0001), while other candidate genes were significantly down-regulated (*Ier2*: 0.93-fold, p<0.05; *Rnaseh2a*: 0.80-fold, p = 0.001; *Tbc1d9*: 0.82-fold, p<0.001; *Lyl2*: 0.72-fold, p<0.001; *Farsa*: 0.66-fold; p<0.0001; *Inpp4b*: 0.54-fold; p<0.0001; and *Tnpo2*: 0.84-fold; p<0.0001). Eight genes also showed similar significant changes in patients with *Escherichia coli* BSI (n = 19) (Fig 4).

#### Four genes are differentially expressed in *S*. *aureus*-challenged human neutrophils

**Fig 5:**
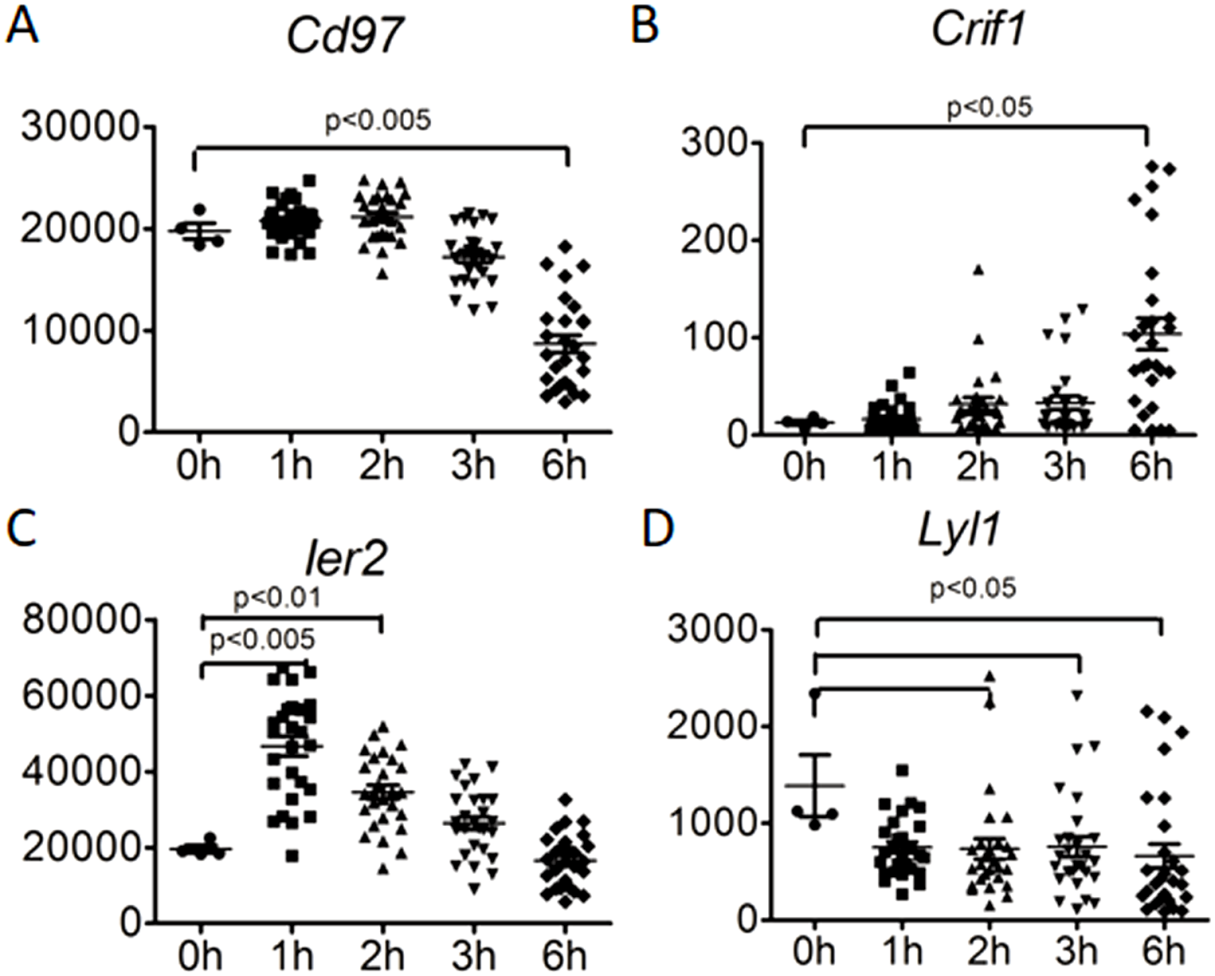
Expression of candidate genes in human neutrophils upon *S*. *aureus* infection. Expression of *Cd97*, *Crif1*, *Ier2* and *Lyl1* in *S*. *aureus*-challenged human neutrophils were significantly different as compared as naïve status. Human neutrophil data from public data set GEO:GSE16837 (http://www.ncbi.nlm.nih.gov/geo/query/acc.cgi?acc=GSE16837) was analyzed. *Cd97* (0.44 fold, p<0.0001) and *Lyl1* (0.48 fold, p<0.05) were decreased at 6hr after *S*. *aureus* stimulation. By contrast, *Crif1* (8.70 fold, p<0.05) and *Ier2* (1.77 fold, p<0.05) were significantly increased at 6hr and 2h, respectively, after *S*. *aureus* stimulation.

Because the neutrophil is the primary host defense cell for management of *S*. *aureus* infection, we next used publically available GEO data to evaluate which genes were differentially expressed in human neutrophils when challenged by *S*. *aureus* (http://www.ncbi.nlm.nih.gov/geo/query/acc.cgi?acc=GSE16837). Of our 11 candidate genes found to be differentially expressed in patients with *S*. *aureus* BSI, four were also significantly differentially expressed in *S*. *aureus*–challenged human neutrophils (*Ier2*, *Crif1*, *Cd97*, and *Lyl1*) (Fig 5). In *S*. *aureus*-challenged human neutrophils, *Cd97* was down-regulated 0.4 fold at 6 hours (p = 0.0015); *Lyl1* was down-regulated 0.5 fold at 2 hours (p = 0.0133), 3 hours (p = 0.0251) and 6 hours (p = 0.0339); *Ier2* was upregulated 2.4 fold at 1 hour (p = 0.0030) and 1.8 fold at 2 hours (p = 0.0056); and *Crif1* was upregulated 8.7 fold at 6 hours (p = 0.0251).

#### Allele specific expression analysis

Four 8-week A/J, C57BL/6J and CSS8 male mice were infected with *S*. *aureus* for 3 hours by intraperitoneal route. White blood cell RNA was extracted and applied to RNA-seq analysis. RNA-seq data was processed using the TrimGalore toolkit[12]. Principal component analysis found the distance difference among the three strains (S2A Fig). For the 11 CSS8 candidate genes, there was fairly even parental origin from either A/J and C57BL/6J (S2B Fig), indicating that the *S*. *aureus* susceptible phenotype was due to multiple gene effect instead of a single candidate gene.

#### *Cd97* has different ligand binding ability between A/J and C57BL/6J

**Fig 6:**
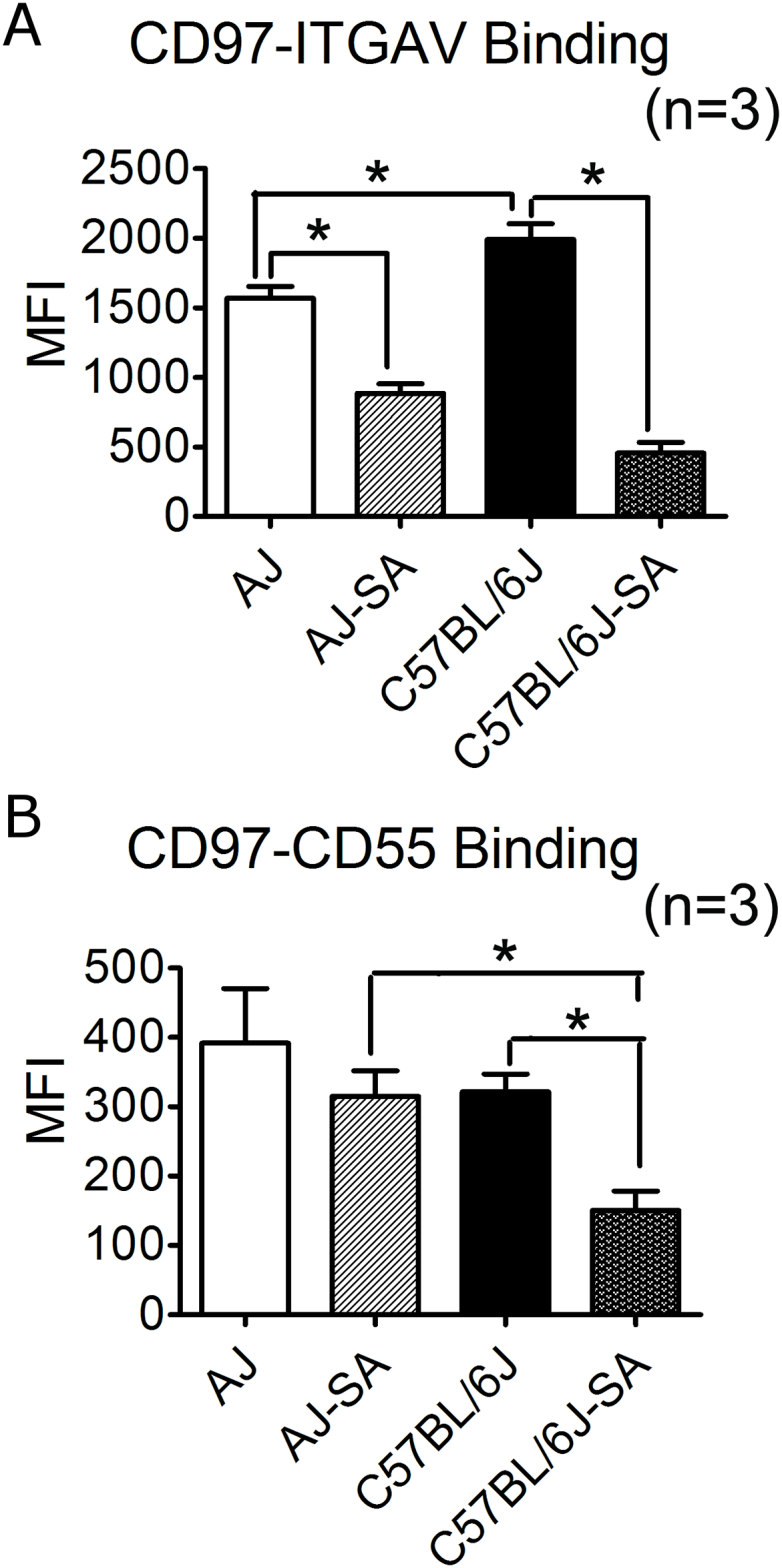
CD97 binding assay. CD97/integrin α5 (ITGAV) and CD97/CD55 binding ability was compared in A/J and C57BL/6J at both naïve and *S*. *aureus* infection condition. **(A)** CD97-Integrin α5 binding assay. At the naïve status, peritoneal neutrophils in both A/J and C57BL/6J (n = 3 in each group) had higher CD97-Integrin α5 binding ability than in the *S*. *aureus* infected condition (p<0.05 in A/J; p<0.05 in C57BL/6J). C57BL/6J peritoneal neutrophils had higher CD97-Integrin α5 binding ability as compared with A/J peritoneal neutrophils (p<0.05). **(B)** CD97-CD55 binding assay. *S*. *aureus* infection reduced CD97-CD55 binding ability in C57BL/6J peritoneal neutrophils (p<0.05) (n = 3 in each group). All mice were 8-week old males.

CD97 is a cell membrane G-protein coupled receptor and its ligand binding affects its biological function. In this study, we focused on CD97/CD55 and CD97/integrin α5β1 binding, as these two are closely associated with host immune function [13, 14]. CD97 from C57BL/6J peritoneal neutrophils had significantly stronger integrin α5β1 binding ability as compared with A/J (Fig 6A), while its binding ability to CD55 was weaker (Fig 6B) (p<0.05 for both). Integrin α5β1 serves as a membrane receptor for matrix fibronectin, which also interacts with fibronectin binding protein (FnBPs) from *S*. *aureus* [15, 16]. The stronger CD97/integrin α5β1 binding in C57BL/6J than in A/J, suggests a more robust host-pathogen interaction in the resistant strain (C57BL/6J) vs the susceptible strain (A/J). The weaker binding of CD97/integrin α5β1 in A/J identified a very important and interesting target for studying host susceptibility to *S*. *aureus*. Because the binding of CD97 and CD55 helps regulate immune cell activation and increase proliferation and cytokine production, the signaling induced by this binding may play a role in the exaggerated cytokine production upon *S*. *aureus* challenge in A/J strain.

#### *Crif1* is consistently expressed in *S*. *aureus* infected mice and humans

**Fig 7:**
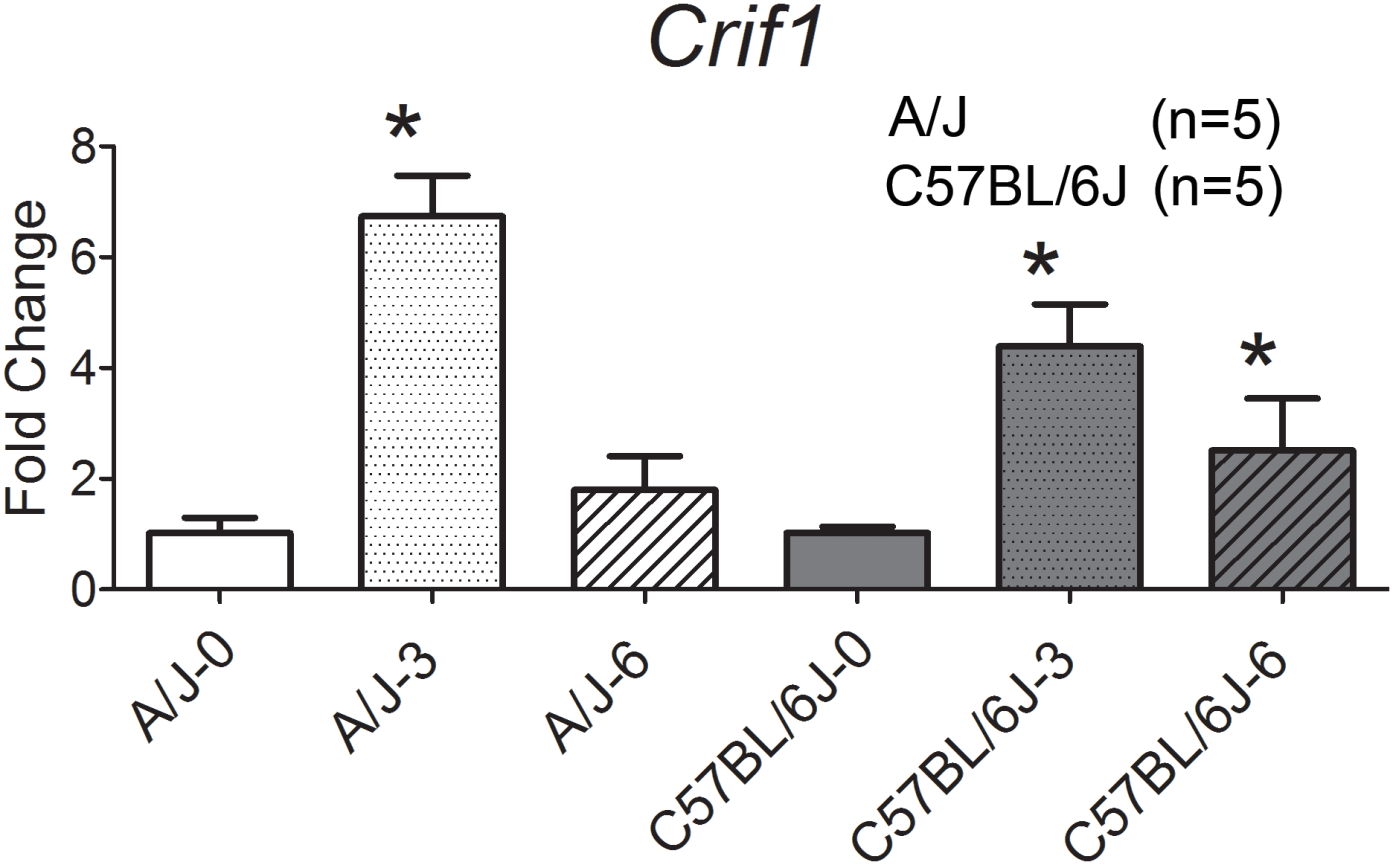
*Crif1* expression is upregulated in *S*. *aureus*-infected A/J and C57BL/6J mice by qPCR. Consistent expression pattern of *Crif1*in A/J and C57BL/6J mouse (n = 5 in each group). *Crif1* showed consistent uninfected vs infected expression patterns between mouse and humans (Figs 4 and 5) At 3 hours and 6 hours post *S*. *aureus* challenge, *Crif1* is upregulated 6.7 fold (p<0.05) and 1.8 fold (p = 0.4) respectively in A/J, and 4.4 fold (p<0.05) and 2.5 fold (p = 0.14) in C57BL/6J. The normalization was conducted within each strain for comparing different time points (e.g. C57BL/6J time points were normalized to C57BL/6J pre-infection time point and A/J time points were normalized to A/J pre-infection time point.) All mice were 8-week old males.

*Crif1* was significantly upregulated in *S*. *aureus*-infected mice (Fig 7), *S*. *aureus*-infected humans (Fig 4), and *S*. *aureus*-challenged human neutrophils (Fig 5). In *S*. *aureus*-infected A/J mice, *Crif1* was upregulated 6.7 fold at 3 hours (p<0.05) and 1.8 fold at 6 hours (p = 0.4) (Fig 7). In *S*. *aureus*-infected C57BL/6J mice, *Crif1* was upregulated 4.4 fold (p<0.05) at 3 hours and 2.5 fold (p = 0.14) at 6 hours (Fig 7). Collectively, these data support *Crif1*’s role in influencing host susceptibility to *S*. *aureus* infection.

#### Apoptosis is increased in A/J bone marrow derived macrophages (BMDM), CSS8 BMDM, and *Crif1* siRNA transfected BMDMs

**Fig 8:**
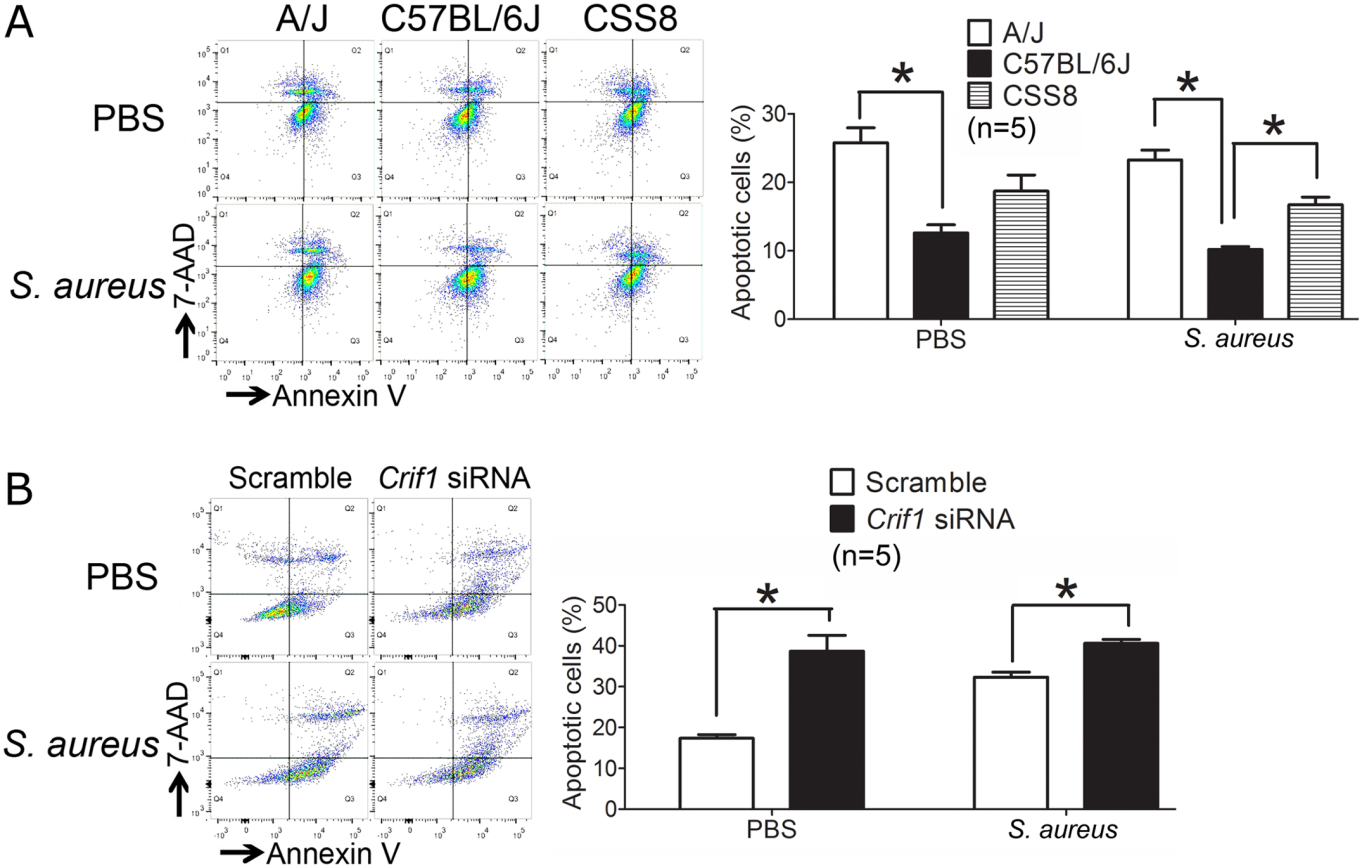
Increased apoptosis is associated with lower *Crif1* expression in A/J and CSS8 macrophages. Apoptosis rates in Figs 8A and 8B cannot be compared because transfection experiments, which intrinsically elicit apoptosis, were performed in (B) but not (A). **(A)** Bone-marrow derived macrophages from A/J and CSS8 demonstrate higher apoptosis level as compared with C57BL/6J in both uninfected (25.8% and 18.7% vs 12.6%; p <0.05) and *S*. *aureus*-challenged conditions (23.3% for A/J, 16.7% for CSS8 and 10.2% for C57BL/6J; p<0.05) (n = 5 in each group). **(B)** Knockdown of *Crif1* by siRNA enhances apoptosis in both uninfected and *S*. *aureus*-challenged conditions. Knockdown of *Crif1* in BMDMs from C57BL/6J mice enhances apoptosis in uninfected status as compared with scramble siRNA (38.7% vs 17.3%; p<0.05). *S*. *aureus* stimulation exhibits similar patterns of apoptosis (40.6% vs 32.3%; p<0.05) (n = 5 in each group). Mice were 8-week old males.

Next, we considered the possible biological basis for the association of *Crif1* expression and susceptibility to *S*. *aureu*s. Given *Crif1’s* key role in regulating apoptosis [17], and the importance of apoptosis in host cellular immunity [18–21], we hypothesized that *Crif1* influenced susceptibility to *S*. *aureus* by increasing apoptosis of host immune cells. To test this hypothesis, we first compared apoptosis of Bone Marrow Derived Macrophages (BMDM) in our susceptible and resistant mice strains. Rates of apoptosis were significantly higher among BMDMs from susceptible mice (A/J and CSS8) as compared to resistant mice (C57BL/6J) in both uninfected (A/J: 25.8%; CSS8: 18.7%; C57BL/6J: 12.6%; p <0.05) and *S*. *aureus*-infected (A/J: 23.3%; CSS8: 16.7%; C57BL/6J: 10.2%; p <0.05) conditions (Fig 8A). Next, we disrupted *Crif1* expression by siRNA transfection of BMDMs. Apoptosis was significantly higher in *Crif1* siRNA knockdown BMDMs as compared to BMDMs transfected with scramble siRNA in both naïve (*Crif1*-knockdown 38.7% vs scramble siRNA 17.3%; p<0.05) and *S*. *aureus*-stimulated conditions (*Crif1*-knockdown 40.6% vs scramble siRNA 32.3%; p<0.05) (Fig 8B). *S*. *aureus* stimulation did not change the apoptosis rate in primary murine (Fig 8A) or siRNA transfected BMDMs (Fig 8B) in our experimental conditions. These findings suggest that reduced *Crif1* expression in A/J and CSS8 mice may contribute to their susceptibility to *S*. *aureus* infection through enhanced cellular apoptosis.

## Discussion

**Fig 9:**
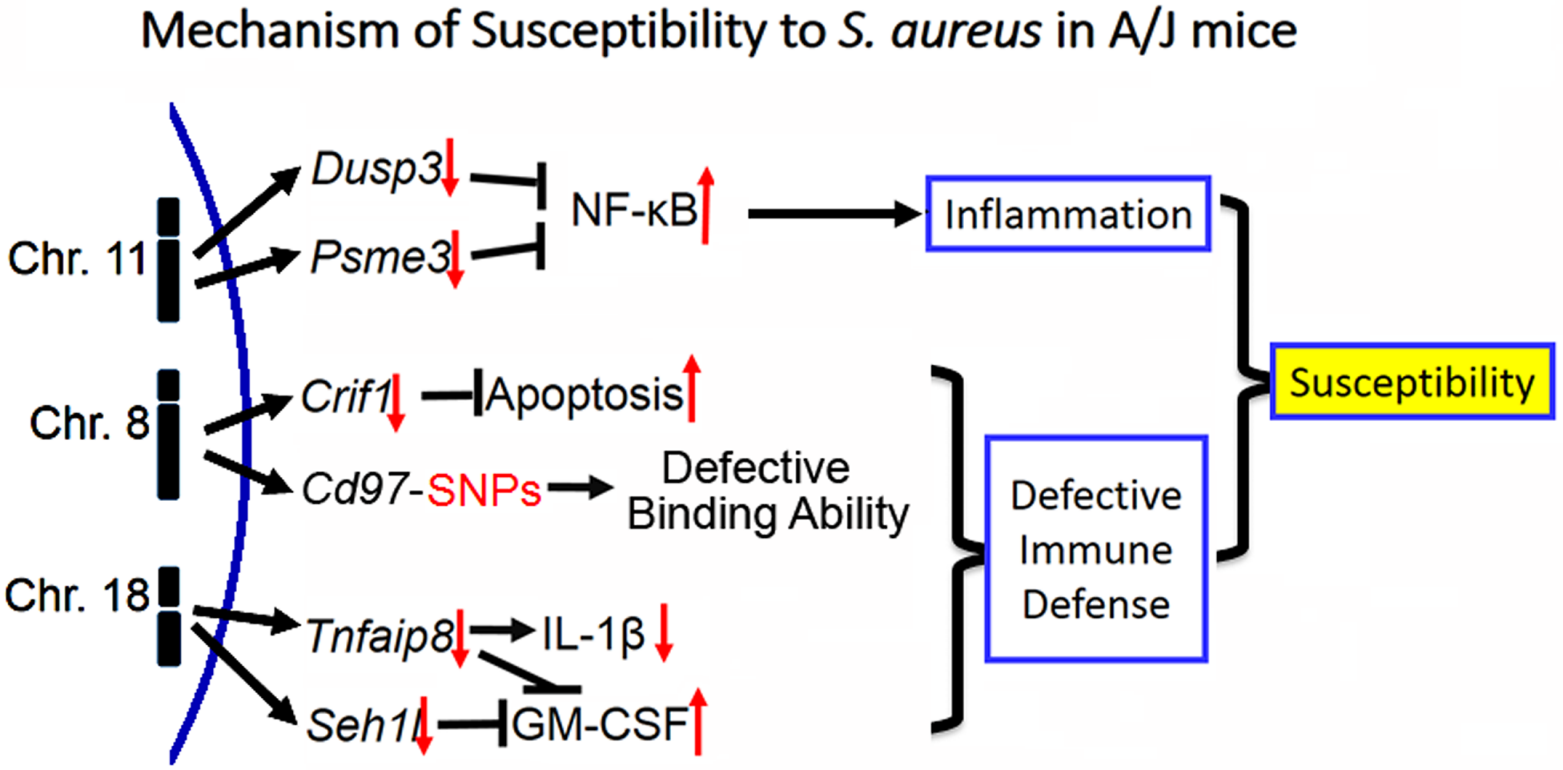
Proposed mechanism of susceptibility to *S*. *aureus* in A/J mice. Summary of candidate gene function in A/J mice responsible for *S*. *aureus* susceptibility. Down-regulation of *Dusp3* and *Psme3* from chromosome 11 enhances inflammation upon *S*. *aureus* infection by activation of NF-κB signaling. Down-regulation of *Crif1* from chromosome 8 compromises host immune defense against *S*. *aureus* by increasing apoptosis. Non-synonymous SNPs in *Cd97* alters ligand binding ability. Down-regulation of *Tnfaip8* and *Seh1l* from chromosome 18 increase the production of GM-CSF and lower expression of *Tnfaip8* decreases the production of IL-1β.

The genetic factors associated with host susceptibility to *S*. *aureus* infection remain largely unknown. Our study overcame the well-known inconsistencies between murine and human sepsis [22] by using a stringent trans-species selection strategy to identify four candidates from murine chromosome 8, and to establish a biological basis for influencing susceptibility for our top two candidates. In this way, *Crif1* and *Cd97* were identified as promising candidate genes associated with *S*. *aureus* susceptibility from murine chromosome 8 (Fig 9).

A robust body of evidence supports our conclusion that *Crif1* contributes to host susceptibility to *S*. *aureus* infection. *Crif1* was under-expressed in susceptible A/J mice as compared with resistant C57BL/6J mice. *Crif1* expression was increased in response to *S*. *aureus* infection in both strains, in *S*. *aureus*-challenged human neutrophils, and in humans with *S*. *aureus* BSI. These findings suggest that *Crif1* is important in host response to *S*. *aureus* and that its comparative under-production in A/J may contribute to that strain’s susceptibility to *S*. *aureus*. Substantial evidence also suggests that the biological basis for *Crif1*’s role in *S*. *aureus* susceptibility may involve apoptois [17]. BMDM from susceptible A/J and CSS8 mice were significantly more apoptotic than BMDM from resistant C57BL/6J strains. siRNA-mediated knockdown of *Crif1* significantly increased apoptosis in both naïve and *S*. *aureus* challenged BMDMs. Because cell survival and death are fundamental parameters in the process of immune function [23–25], *Crif1* may be involved in host immune defense against *S*. *aureus* in both humans and mice.

The functional variety of *Crif1* requires further detailed investigation on its biological relevance to *S*. *aureus* susceptibility. *Crif1* plays a key role in mitochondrial homeostasis of host cell and oxidative phosphorylation. *Crif1* is involved in regulation of oxidative phosphorylation and respiration by lymphocyte expansion molecule (LEM) to promote antigen-dependent CD8(+) T cell proliferation [26] and by lymphocyte-specific protein tyrosine kinase (Lck) to cause blood malignancies [27]. The fundamental effect of *Crif1* on mitochondrial function is not limited to the immune system [28]. For example, reduced expression of *Crif1* has been shown to play an important role in Alzheimer’s disease through regulation of Aβ-induced mitochondrial disruption [29], and *Crif1* deficiency reduces adipose OXPHOS capacity and triggers inflammation and insulin resistance in mice [30]. Disruption of *Crif1* in mouse islet beta cells leads to mitochondrial diabetes with progressive beta cell failure [31]. Finally, cardiomyocyte specific deletion of *Crif1* causes mitochondrial cardiomyopathy in mice [32]. Collectively, these reports of *Crif1*’s effect on mitochondrial function suggest that its impact on susceptibility to *S*. *aureus* may occur in part by influencing central energy metabolism in the host.

*Crif1* is also involved in major signaling pathways to modulate cell fate and functions. *Crif1* affects PKA/CREB signaling pathway to promote adipogenic differentiation of bone marrow mesenchymal stem cells [33]. As PKA/CREB signaling is also fundamentally involved in different immune cells, *Crif1* may indirectly affect host immune defense against *S*. *aureus* in both the innate [34–36] and adaptive immune systems [37, 38]. For example, *Crif1* is a novel transcriptional coactivator of STAT3 [17], a critical component of many cytokine receptor systems involved in pathogen resistance [39–41]. Interestingly, mutations in STAT3 cause Job’s syndrome, which is characterized by recurrent *S*. *aureus* infections and hyper-IgE production [42, 43]. The current discovery suggests that *Crif1* may indirectly affect *S*. *aureus* susceptibility through an autocrine or paracrine signaling pathway by affecting the JAK-STAT responsiveness to cytokines. Further, *Crif1* is required in RNA interference and Dicer-2 stability [44] which are vital parts of host immune response to viruses and other foreign genetic material [45, 46].

*Cd97* is a transmembrane G-Protein Coupled Receptor, characterized by an extended extracellular region to mediate cell-cell adhesion and interaction [47]. The function of *Cd97* is critical for host immune defense, and upon activation lymphoid, myeloid cells and neutrophils increase *Cd97* level to promote adhesion and migration [48]. The ligand binding of *Cd97* initiates several important biological functions. For example, the binding between CD97 and CD55 has been shown to regulate granulocyte homeostasis [49], T-cell activation, proliferation and cytokine production [50, 51]. Interestingly, CD97 also binds to integrin α5β1, a cell surface fironection receptor, to regulate inflammatory cytokine production [14]. Because fibronection binding proteins are important mediators of *S*. *aureus* pathogenesis, the involvement of CD97 bridges the host and *S*. *aureus* interaction and serves as an important future target. Our flow cytometry study showed that peritoneal neutrophils from C57BL/6J have less CD55 binding than A/J, which indicates that the SNP of CD97 may predispose A/J to exaggerated cytokine production upon *S*. *aureus* challenge, rendering A/J mice susceptible to *S*. *aureus* infection. While on the other hand, the interaction between CD97 and integrin α5β1, the matrix fibronectin receptor, was enhanced in C57BL/6J, indicating that cells from C57BL/6J may have stronger capability to trap *S*. *aureus* through CD97-integrin α5β1-fibronetin-fibronection binding protein complex during *S*. *aureus* infection, providing a very promising candidate to study host- *S*. *aureus* interaction. Our future direction will mainly focus on the study of *Cd97* function of initiating host immune defense against *S*. *aureus*.

Considerably less is known about the potential role of our remaining candidate genes, *Ier2* and *Lyl1*, in determining host susceptibility to *S*. *aureus*. *Ier2* is an immediate early response gene, affecting cell adhesion and motility [52, 53]. Although it has some DNA binding ability [54], nothing is known regarding its involvement in immunity or host defense. Lymphoblastomic leukemia 1 (*Lyl1*) is a transcription factor involved in hematopoietic stem cell function [55], and B cell differentiation [56]. The overexpression of *Ly11* induces T- and B-cell lymphoma in mice [57]. We suspect that the function of *Lyl1* in *S*. *aureus* susceptibility is mainly relevant to T- and B- cell. Although we were unable to evaluate biological plausibility for *Lyl1* and *Ier2* because of limited prior information about their role in immune function, future studies could expand our understanding of their role in this area. Thus, it is important that they not be ruled out as potential candidate genes.

The current study has limitations. First, it is possible that multiple genes within the QTL may contribute to the phenotype of interest. This possibility is supported by the robust number of differentially expressed genes in the QTL region, by the allele-specific expression analysis, and by our discovery of several potential candidate genes. Second, the essential function maintaining mitochondrial homeostasis of host cell and oxidative phosphorylation [26, 30, 32] provides the possibility that *Crif1* is merely a general host responsive factor coping with stress during inflammation. Third, *S*. *aureus* colonization can increase a patient’s risk for subsequent *S*. *aureus* infection[58], and we did not limit control subjects to those who were colonized with *S*. *aureus*. However, given the fact that approximately one-third of all persons are colonized with *S*. *aureus*[58], it is likely that a many of our uninfected control subjects were in fact colonized with *S*. *aureus*. Fourth, the murine sepsis model can not fully address the full diversity of disease caused by *S*. *aureus*, such as skin and soft tissue infection, osteomyelitis and endocarditis [59, 60]. Thus, our consideration of survival as a dichotomous trait is likely to overly simplify the full complexity of susceptibility to bacterial infection. Alternately, our approach may disregard genes that contribute in combination to *S*. *aureus* susceptibility. Our current model of interaction for the identified S. *aureus* susceptibility genes on the three chromosomes is illustrated in Fig 9. To ultimately solve these limitations and mechanistically understand the biological relevance of candidate genes, additional experiments are underway in our lab, including defining the pathogenesis of *Crif1*, *Dusp3*, and *Tnfaip8* using knockout mice. Fifth, our approach did not include the role of insertions/deletions in coding regions.

A growing number of studies have evaluated genetic susceptibility to *S*. *aureus* infections in humans. We reported a case-control Genome-wide Association study (GWAS) of 361 patients with SAB who were matched to 699 controls[61]. Ye et al reported a similar design with an outcome of any *S*. *aureus* infection (309 cases, 2925 controls)[62]. Neither identified SNPs reaching genome-wide significance, probably due to relatively small sample size. More recently, however, we have identified genetic variants within the HLA class II region in two distinct study populations that were associated with increased susceptibility to *S*. *aureus* infection at a level of genome-wide significance. Using a population of over 50,000 White subjects (4701 cases with *S*. *aureus* infection and 45,344 matched controls), we identified two imputed SNPs near HLA-DRA and HLA-DRB1 genes that were genome wide significant (rs115231074: p = 1,3 x 10^−10^ and rs35079132: p = 3.8 x 10^−8^) and one genotyped SNP that almost achieved genome-wide significance (rs4321864: p = 8.8 x 10^−8^).[63] Finally, we used admixture mapping to evaluate the impact of genetic variation on susceptibility to *S*. *aureus* infection in a cohort of African-Americans with SAB.[64] After empirical multiplicity adjustment, a single region in HLA class II was found to exhibit a genome-wide statistically significant increase in European ancestry, providing additional evidence for genetic variation influencing HLA-mediated immunity. Taken together with the findings of the current manuscript, it is likely that genetic susceptibility to *S*. *aureus* infection is complex and syndrome-specific.[65] Thus, the genetic variation found to be important in *S*. *aureus* sepsis may differ from that influencing pneumonia, soft tissue infection, or endocarditis.

Despite our study’s limitations, the present investigation makes several key observations. First, we have identified one QTL on chromosome 8 that is significantly linked to survival after infection with *S*. *aureus*. Among the 102 genes in the QTL that was associated with susceptibility to *S*. *aureus*, four show evidence of association in both *S*. *aureus*-infected mice and humans. Of these 4 genes, *Crif1* and *Cd97* also exhibit biological evidence for their relevance in *S*. *aureus* infection. *Crif1* exhibited differential expression between naïve and *S*. *aureus*-infected mice; differential expression between susceptible and resistant mice; and had human orthologues that exhibited a consistent pattern of expression in patients with *S*. *aureus* BSI and in human neutrophils challenged with *S*. *aureus*. Biologically, several lines of evidence suggest that *Crif1* influences susceptibility to *S*. *aureus* by apoptosis of host defense cells. *Cd97* has damaging SNPs in C57BL/6J and had significant human orthologue expression in patients with *S*. *aureus* BSI. Ligand binding assay also shows the stronger CD97/integrin α5 binding ability in resistant strain but not in susceptible strain. Collectively, our results support a potential role of *Crif1* and *Cd97* in host response to *S*. *aureus* by affecting host cell fate during inflammation caused by *S*. *aureus*.

## Materials and methods

### Ethics statement

All animal experiments were approved by the Institutional Animal Care and Use Committee (IACUC Protocols A191-12-07/143-15-05) of Duke University. The most current edition of the Guide For The Care And Use of Laboratory Animals was followed when developing SOPs and policies. The the human studies referenced in this work were approved by Duke University Medical Center Institutional Review Board (Durham, NC), Durham VA Medical Center Institutional Review Board (Durham NC), and Henry Ford Hospital Institutional Review Board (Detroit MI). Written informed consent was obtained for all human subjects.

### Human subjects

Subjects were enrolled at Duke University Medical Center (DUMC; Durham, NC), Durham VAMC (Durham, NC), and Henry Ford Hospital (Detroit, Michigan) as part of a prospective, NIH-sponsored study to develop novel diagnostic tests for severe sepsis and community-acquired pneumonia as mentioned before [66–68]. All participants were adults. Detailed clinical information about these patients, including age and gender, has been previously published [11]. RNA was obtained from blood drawn at the time patients initially presented to the Emergency Department with sepsis. RNA expression data from patients who were ultimately found to have BSI with either *S*. *aureus* (n = 32) or *E*. *coli* (n = 19) were used in this study. Healthy controls were defined as uninfected human (n = 44), enrolled as part of a study on the effect of aspirin on platelet function among healthy volunteers [69]. Subjects were recruited through advertisements posted on the Duke campus. Blood used to derive gene expression data in these healthy controls was drawn prior to aspirin challenge. Human orthologs of murine genes were identified by Chip comparer (http://chipcomparer.genome.duke.edu/) as reported before [11]. When there were multiple orthologs, we preferentially used the anti-sense target probes that shared the fewest probes with other genes as identified by the probe label.

### Mouse strains

C57BL/6J, A/J, and CSS8 mice were purchased from the Jackson Laboratory (Bar Harbor, ME). All the mice were allowed to acclimate for more than 7 days before experiments. For generation of F1 progeny, CSS8 mice were mated with C57BL/6J in reciprocal crosses [C57BL/6J male × CSS8 female and C57BL/6J female × CSS8 male] to generate an F1 population with heterozygous chromosome 8. To generate N2 backcross mice for QTL linkage analysis, F1 (C8A) mice were backcrossed with C57BL/6J to produce a total of 337 progeny that were used for phenotyping. Specific numbers of mice employed in experiments are provided in the tables and figures presenting the data.

### Preparation of bacteria

*S*. *aureus* clinical strain, Sanger 476 was used in the mortality and infection studies. For preparation of *S*. *aureus* for injection, overnight culture of *S*. *aureus* was diluted 100 folds with fresh tryptic soy broth (TSB) and shake at 37°C with aeration to log-phase (OD600 ≈ 0.8). *S*. *aureus* was harvested by centrifugation at 3000rpm for 10 minutes at 4°C, washed once in DPBS and re-suspended in DPBS.

### Murine sepsis experiment and bacterial load quantification

For murine peritonitis-sepsis experiments, 8-week-old male mice (n = 8) in each strain of C57BL/6J, A/J, and CSS8 were injected via intraperitoneal route with 10^7^CFU/g *S*. *aureus* (Sanger 476) or 2×10^5^ CFU/g *E*. *coli* (K1H7) and observed every 6 hours for morbidity continuously for 5 days.

### QTL linkage analysis

Polymorphic microsatellite markers on chromosome 8 between C57BL/6J and A/J were chosen from a database maintained by Mouse Genomic Informatics (http://www.informatics.jax.org/). Seventeen microsatellite markers were selected with an average inter-marker distance of 0.65 cM covering chromosome 8. A total of 337 N2 backcross mice were generated, all of which were genotyped for each microsatellite marker by PCR amplification and gel electrophoresis. J/qtl software was used to analyze phenotype and genotype data for linkage of survival time after infection with *S*. *aureus* Sanger 476 and marker location. Phenotypes were defined as either sensitive or resistant based on the dichotomization of survival data (survival of less than 2 day is “0” and survival of longer than 2 days is “1”, respectively). All linkage analysis results were expressed as LOD scores. LOD score was considered “suggestive” if > = 0.43 (p = 0.63) and “significant” if > = 1.51 (p = 0.05). Threshold values for linkage were determined by a 1,000 permutation test by using J/qtl.

### Microarray

Accession numbers for murine genes and their human orthologs were identified in NCBI and are provided in Table 1. The microarray data have been deposited in the NCBI GEO and are accessible through GEO series accession no. GSE19668 [6]. RNA integrity numbers (RIN) were calculated for all samples and found to be within tolerance limits (RIN > 7) (S3 Table). Post-processing of microarray data included several steps. The microarray gene expression data was analyzed using Partek Genomic Suite 6.4 software (Partek Inc., Louis, MO). All Affymetrix CEL files were imported and normalized using robust multiarray averaging (RMA). *Analysis* was performed using Analysis of Variance (ANOVA) and multi-test correction for p-values in Partek Genomic Suite. Differentially expressed genes between susceptible A/J and resistant C57BL/6J were identified a) at all pre-infection and post-infection time points (for Strategy 2) and b) only in *S*. *aureus* infection state (for Strategy 1). Student’s t-test was used to test for differential expression between 2 groups (eg, AJ mice at time 0 vs. time 2 hours, etc.). The lists of significant differentially expressed genes were generated based on a criterion of ≥2 relative fold change at a false discovery rate (FDR) of ≤ 5% as previously described[70].

**Table 1:** Accession numbers.

Genes	Gene ID from NCBI (murine)	Gene ID from NCBI (human)
*Asf1b*	66929	55723
*Cd97*	26364	976
*Crif1*	102060	90480
*D8Ertd738e*	101966	28974
*Dnaja2*	56445	10294
*Farsa*	66590	2193
*Hook2*	170833	29911
*Ier2*	15936	9592
*Inppb4*	234515	8821
*JunB*	16477	3726
*Klf1*	16596	10661
*Lyl1*	17095	4066
*Mylk3*	213435	91807
*Mri1*	67873	84245
*Nfix*	18032	4784
*Olfr370*	258267	-
*Phkb*	102093	5257
*Pkn1*	320795	5585
*Prdx2*	21672	7001
*Prkaca*	18747	5566
*Rad23a*	19358	5886
*Rnaseh2a*	69724	10535
*Syce2*	71846	256126
*Tbc1d9*	71310	23158
*Tnpo2*	212999	30000
*Zfp423*	94187	23090

### Quantitative PCR

Blood was obtained by cardiac puncture from all mice strains. Total RNA was isolated using RNeasy kits (Qiagen) primed with random hexamer oligonucleotides and reversely transcribed using Invitrogen SuperScript II. Real-time quantitative PCR was performed using SYBR Green Mastermix (ABI). The qPCR primers for candidate genes are provided in S4 Table. All data were normalized to 18s rRNA (S5 and S6 Tables).

### Flow cytometry analysis of apoptosis

Bone marrow-derived macrophages (BMDMs) were differentiated from male A/J, C57BL/6J and CSS8 as before [8]. 2 × 10^6^ BMDMs were seeded to 6-well plate and cultured overnight. On the next day, PBS with *S*. *aureus* or same volume of PBS was added to BMDMs at MOI 10 and cultured for 1 hour. After washing twice with PBS, BMDMs were detached from plate by EDTA [71] and stained with FITC-Annexin-V and 7-AAD for 20 minutes before analysis through BD FACSCanto II. Double positive of Annexin-V and 7-AAD were determined as late apoptotic cells and further analyzed for their apoptosis rate.

### Small interfering RNA (siRNA) experiments

siRNAs were purchased from Invitrogen and transfected into BMDMs of C57BL/6J by Lipofectamine RNAiMAX (Invitrogen) according to the manufacturer’s instructions as before [8]. Twenty-four hours post-transfection, cells were infected with *S*. *aureus* by MOI 10 for 1 hour and further analyzed of apoptosis. In parallel experiments, cells were harvested for RNA and qPCR analysis for their level of candidate genes. A full list of gene names and siRNA ID numbers are listed in S7 Table. Knockdown efficiency of SiRNA is shown in S3 Fig.

### QTL region SNP functional analysis

The 1505 version of the Sanger Mouse Genomes Project sequence variation tool was used to identify all known non-synonymous sequence variants for the genes in the QTL region for the A/J and C57BL/6NJ mouse strains. This analysis utilized the C57BL/6J mouse strain as a reference to identify variants and was carried out at1 kbp resolution. These SNPs were then processed through the S.I.F.T. (Sorting Intolerant From Tolerant) program which is a platform that can be used to predict whether a specific amino acid substitution is a functionally damaging alteration [72]. Analyses for these non-synonymous SNPs were done using the program’s default threshold settings (cutoff = 0.05).

### CD97 ligand binding assay

BD FACSCanto was used to evaluate ligand binding. Mouse peritoneal cells with or without *S*. *aureus* challenge were harvested and incubated with a mixture of recombinant mouse CD55 protein (Thermo Fisher) plus phycoerythrin-conjugated anti-mouse CD55 (BioLegend); or recombinant mouse integrin α5β1 (R&D Systems) plus Alexa Fluo 488-conjugated anti-mouse CD49e (α5) (BioLegend) for 30 minutes at room temperature following manufacturer’s instruction. Cells were assayed using the respective fluorescence channel.

### RNA-seq

RNA quality and concentration were assessed with a Fragment Analyzer (Advanced Analytical) and Qubit 2.0 (ThermoFisher Scientific). For each sample, two hundred nanograms of total RNA was used for library construction. poly(A) mRNA capture and construction of stranded mRNA-seq libraries from intact total RNA (RIN numbers >7) was achieved using the commercially available KAPA Stranded mRNA-Seq library preparation Kit. In brief, mRNA transcripts were first captured using magnetic oligo-dT beads, fragmented using heat and magnesium, and reverse transcribed to produce dscDNA. Illumina standard sequencing adapters were then ligated to the dscDNA fragments and amplified to produce the final RNA-seq library. Libraries were indexed using a molecular indexing approach allowing for multiple libraries to be pooled and sequenced on the same sequencing lane on a HiSeq 4000 Illumina sequencing platform. After quality check of each individual library, the indexed libraries were diluted to 10nM, pooled at equimolar ratios and sequenced on 2 lanes of HiSeq 4000 with 50bp Single Read protocol. Data was demultiplexed and Fastq files were generated using BcltoFastq 2.19 script provided by Illumina.

### RNA-seq data analysis

RNA-seq data was processed using the TrimGalore toolkit[12], which employs Cutadapt[73] to trim low quality bases and Illumina sequencing adapters from the 3’ end of the reads. Only reads that were 20nt or longer after trimming were kept for further analysis. Reads were mapped to the GRCm38v73 version of the mouse genome and transcriptome[74] using the STAR RNA-seq alignment tool[75]. Reads were kept for subsequent analysis if they mapped to a single genomic location. Gene counts were compiled using the HTSeq tool [76]. Only genes that had at least 10 reads in any given library were used in subsequent analysis. Normalization and differential expression was carried out using the DESeq2[77] Bioconductor[78] package with the R statistical programming environment[79]. We included batch and sex as cofactors in the differential expression model. The false discovery rate was calculated to control for multiple hypothesis testing. Gene set enrichment analysis[80] was performed to identify differentially regulated pathways and gene ontology terms for each of the comparisons performed.

### Statistical analyses

Group differences in the distributions of continuous measures for candidate gene expression at times 0 and 2 hours were tested with Student’s T-test and the distributions for apoptosis were evaluated with the Mann Whitney-U test. The primary question of interest for the septic human cohort gene expression data was the presence of differences in the *S*. *aureus*-infected patients. For this reason, pairwise comparisons were made between healthy control subjects and either patients with *S*. *aureus* BSI or *E*. *coli* BSI. Differences in survival times of mice were examined with Kaplan-Meier plots and statistical differences in survival across different mouse strains were tested with the log-rank test. P-values smaller than 0.05 were considered statistically significant.

## Supporting information

S1 Fig Kaplan-Meier curve of *E*. *coli* intraperitoneal sepsis (n = 8 in each group). Mice were 8-week old males.
(TIF)

S2 Fig Allele-specific expression analysis by RNA-seq of A/J, C57BL/6J, and F1 (CSS8 x C57BL/6J). **(A)** PCA-plot of A/J, C57BL/6J and F1 (CSS8 x C57BL/6J). Principal component analysis for RNA-seq data. **(B)** Allele specific expression of the 11 candidate genes. For the 11 candidate genes in A/J chromosome 8 QTL, an even distribution of parental origins was observed in the F1 (CSS8 x C57BL/6J). (N = 4 male mice [8 week age] for each group.)
(TIF)

S3 Fig Knockdown efficiency of candidate genes. (TIF)

S1 Table Genes exhibiting significantly different expression in response to *S*. *aureus* infection in A/J vs. C57BL/6J by microarray. Expression data are provided for all 8 genes that were significantly differentially expressed in A/J but not C57BL/6J. No genes were significantly differentially expressed at 0 vs. 2 hours in only C57BL/6J. Multiple comparisons adjustments were applied using False Discovery Rates of ≤ 5%. 8-week old male A/J mice (n = 5 in each group) were used for experiments.
(TIF)

S2 Table Genes significantly differentially expressed between A/J and C57BL/6J mice at 0, 2, 4, 6, and 12 hours after infection with *S*. *aureus*. Data previously published in S Table 1 of Ahn et al (https://doi.org/10.1371/journal.ppat.1001088.s007) [6].
(TIF)

S3 Table RIN scores of microarray samples. (TIF)

S4 Table List of qPCR primers for candidate genes. (TIF)

S5 Table **A.** Ct values for qPCR results of 5 candidate genes identified by Strategy 1. Male 8 week-old mice were used (n = 4 in each group). **B.** 18s rRNA normalized Ct values for qPCR results of 5 candidate genes identified by Strategy 1. Male 8 week-old mice were used (n = 4 in each group).
(TIFF)

S6 Table **A.** Ct values for qPCR results of 6 candidate genes identified by Strategy 2. Male 8 week-old mice were used (n = 5 in each group). **B.** 18s rRNA normalized Ct values for qPCR results of 6 candidate genes identified by Strategy 2. Male 8 week-old mice were used (n = 5 in each group).
(TIFF)

S7 Table List of siRNAs used in this study. (TIF)
