# Supplementary figures and images for: Candidate genes on murine chromosome 8 are associated with susceptibility to Staphylococcus aureus infection in mice and are involved with Staphylococcus aureus septicemia in humans
Source: PLoS One. 2017 Jun 8;12(6):e0179033. doi: 10.1371/journal.pone.0179033 (PMC5464679; doi:10.1371/journal.pone.0179033)

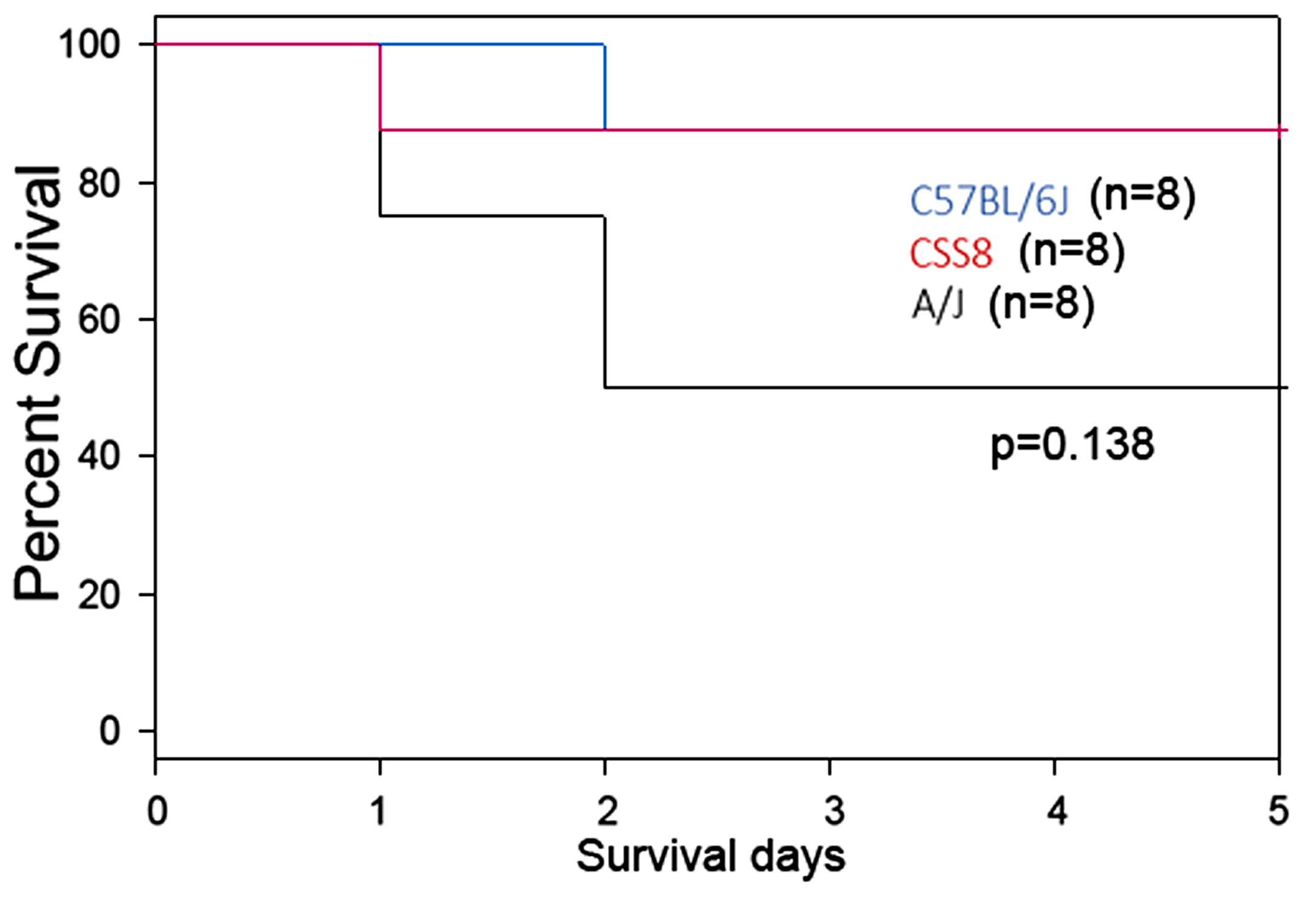

Supplement: S1 Fig — Mice were 8-week old males. (TIF) [file pone.0179033.s001.tif]

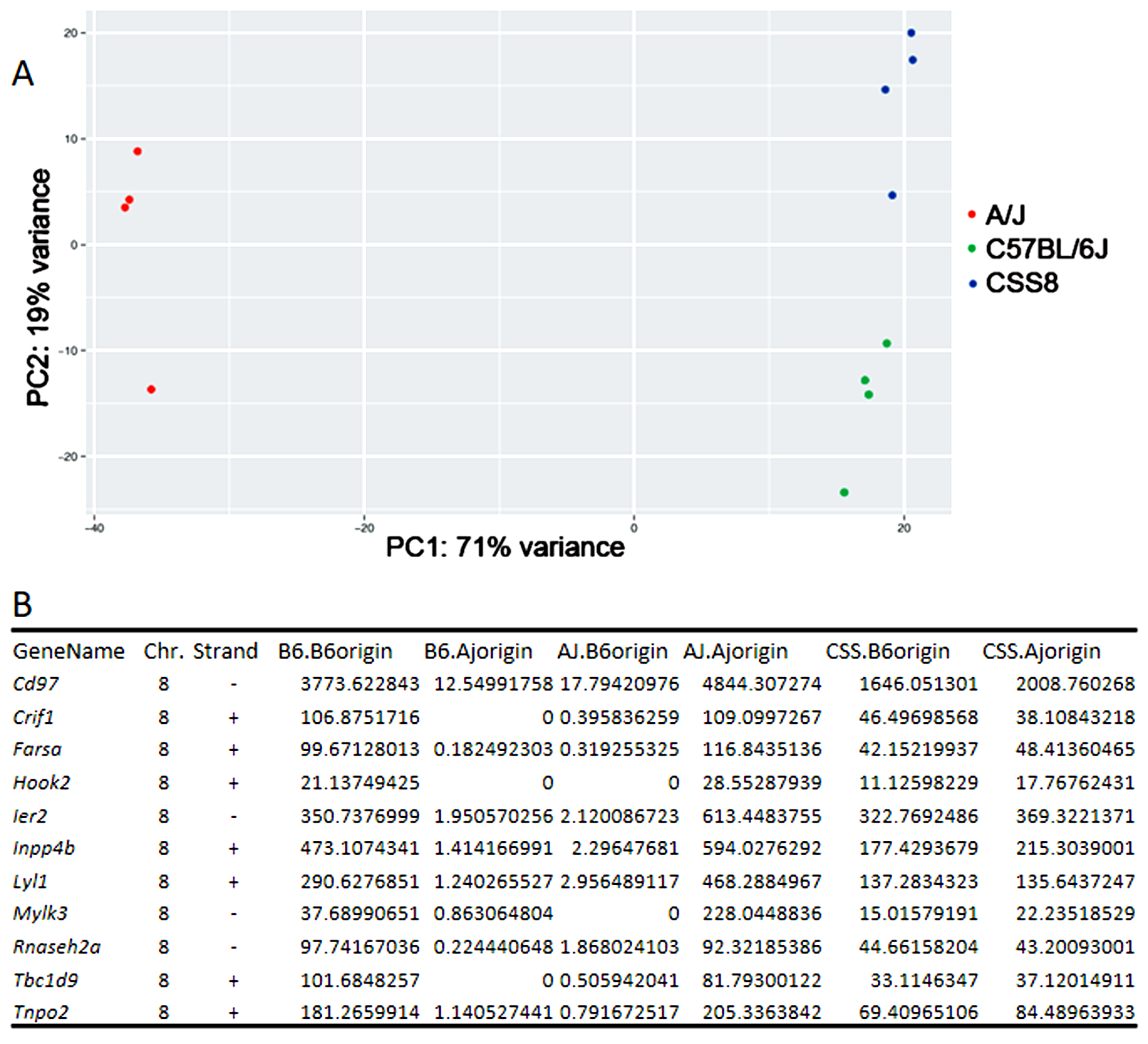

Supplement: S2 Fig — (A) PCA-plot of A/J, C57BL/6J and F1 (CSS8 x C57BL/6J). Principal component analysis for RNA-seq data. (B) Allele specific expression of the 11 candidate genes. For the 11 candidate genes in A/J chromosome 8 QTL, an even distribution of parental origins was observed in the F1 (CSS8 x C57BL/6J). (N = 4 male mice [8 week age] for each group.) (TIF) [file pone.0179033.s002.tif]

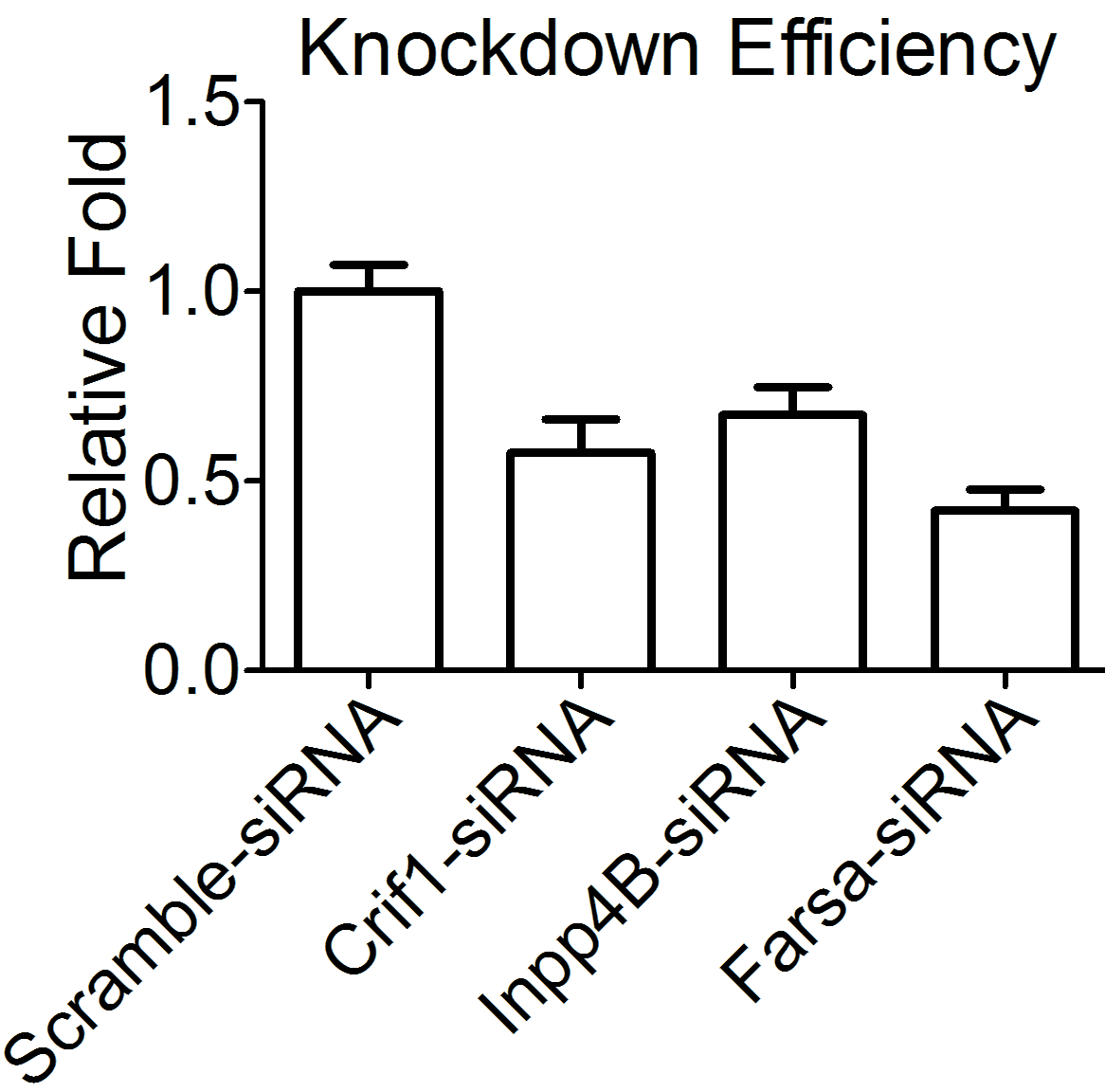

Supplement: S3 Fig — (TIF) [file pone.0179033.s003.tif]

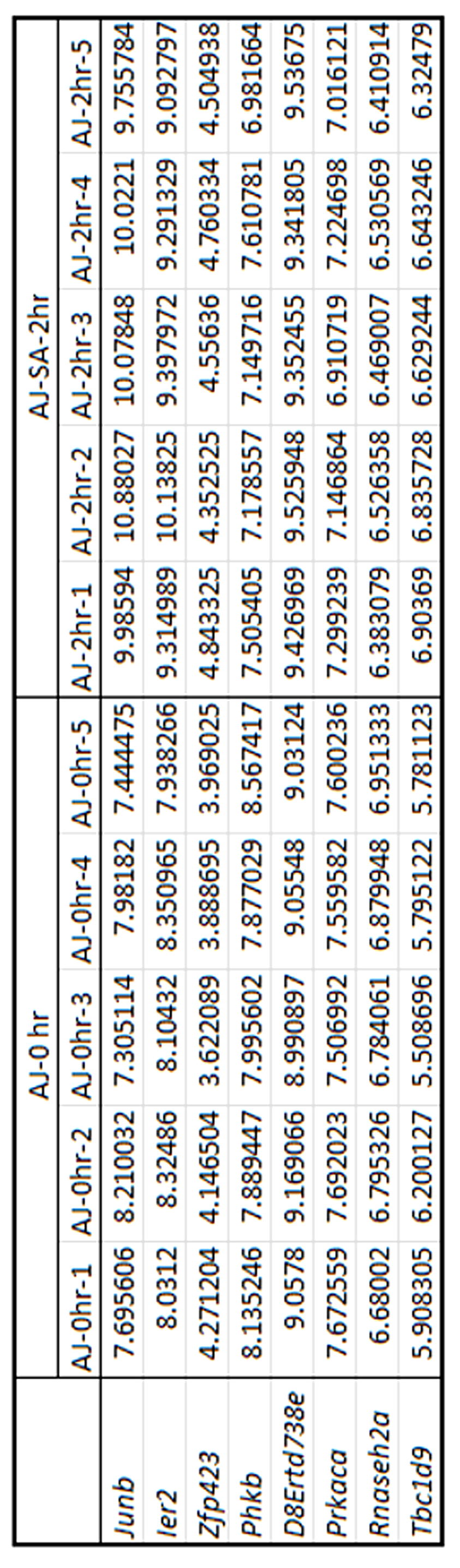

Supplement: S1 Table — Expression data are provided for all 8 genes that were significantly differentially expressed in A/J but not C57BL/6J. No genes were significantly differentially expressed at 0 vs. 2 hours in only C57BL/6J. Multiple comparisons adjustments were applied using False Discovery Rates of ≤ 5%. 8-week old male A/J mice (n = 5 in each group) were used for experiments. (TIF) [file pone.0179033.s004.tif]

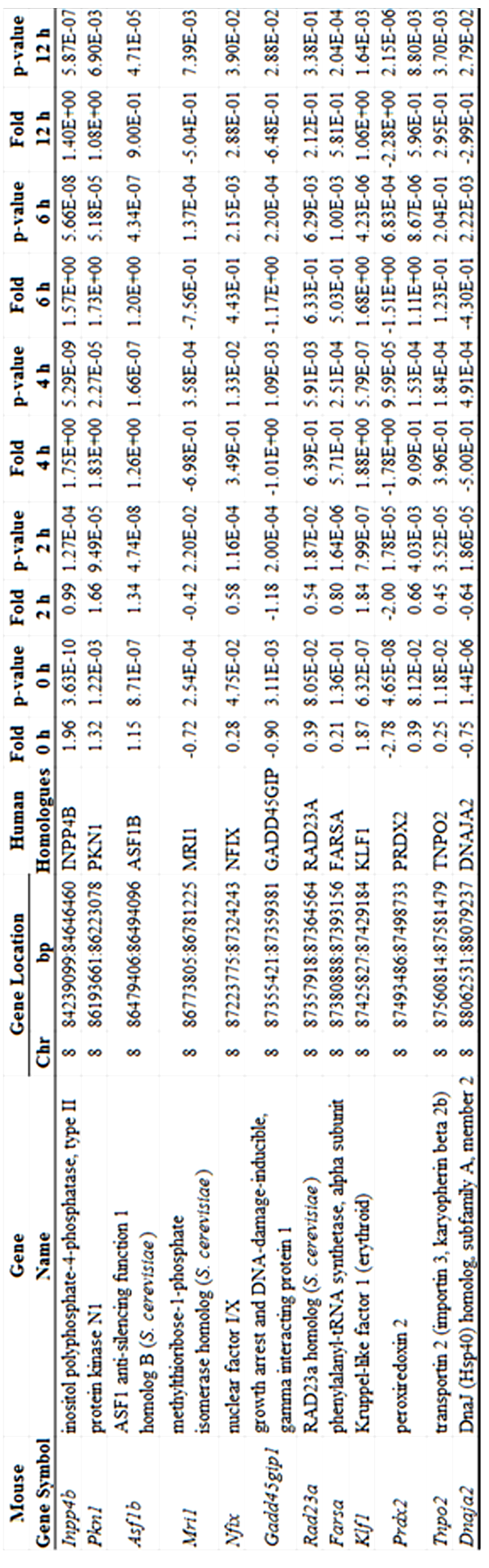

Supplement: S2 Table — Data previously published in S Table 1 of Ahn et al (https://doi.org/10.1371/journal.ppat.1001088.s007) [6]. (TIF) [file pone.0179033.s005.tif]

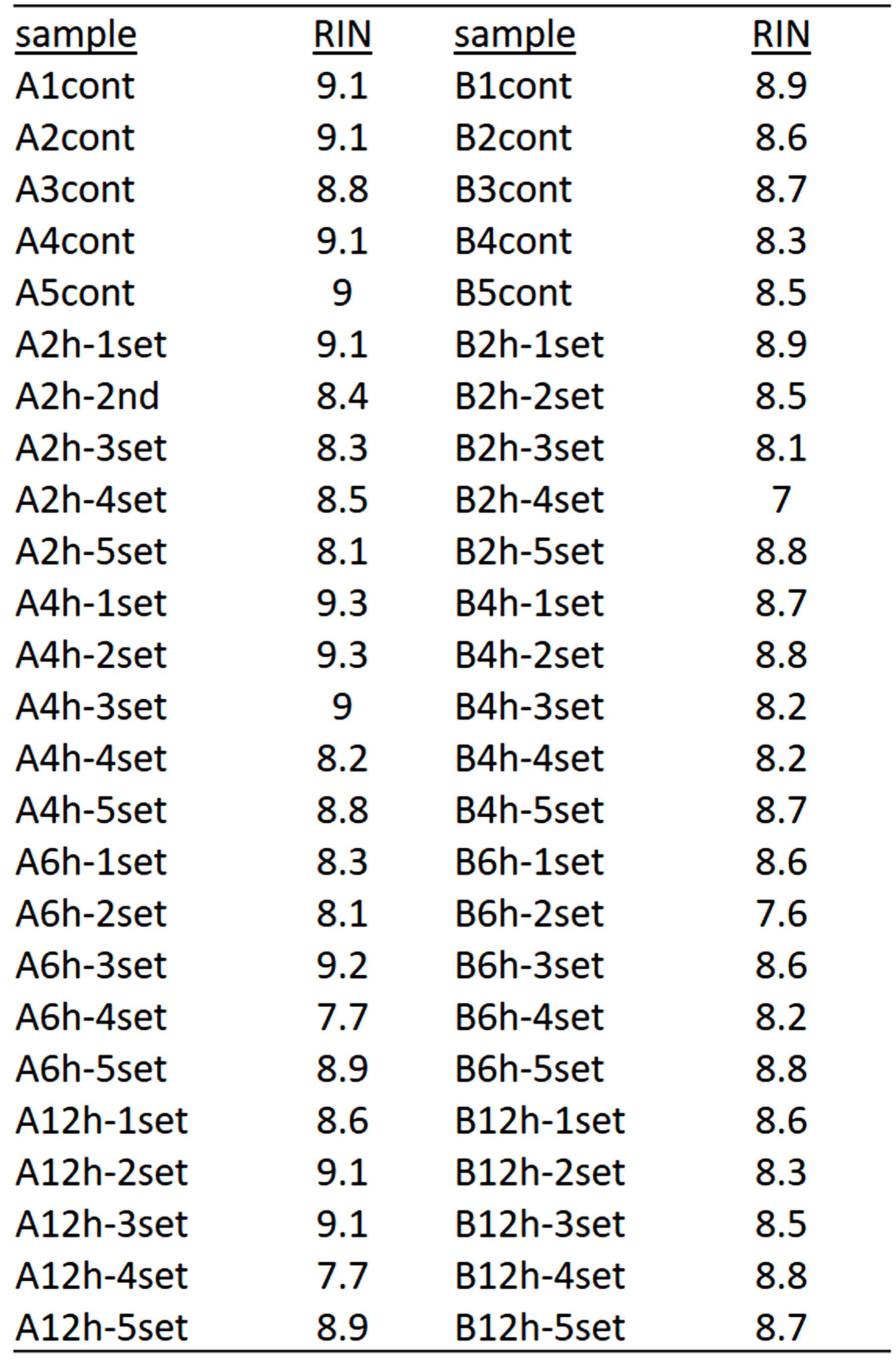

Supplement: S3 Table — (TIF) [file pone.0179033.s006.tif]

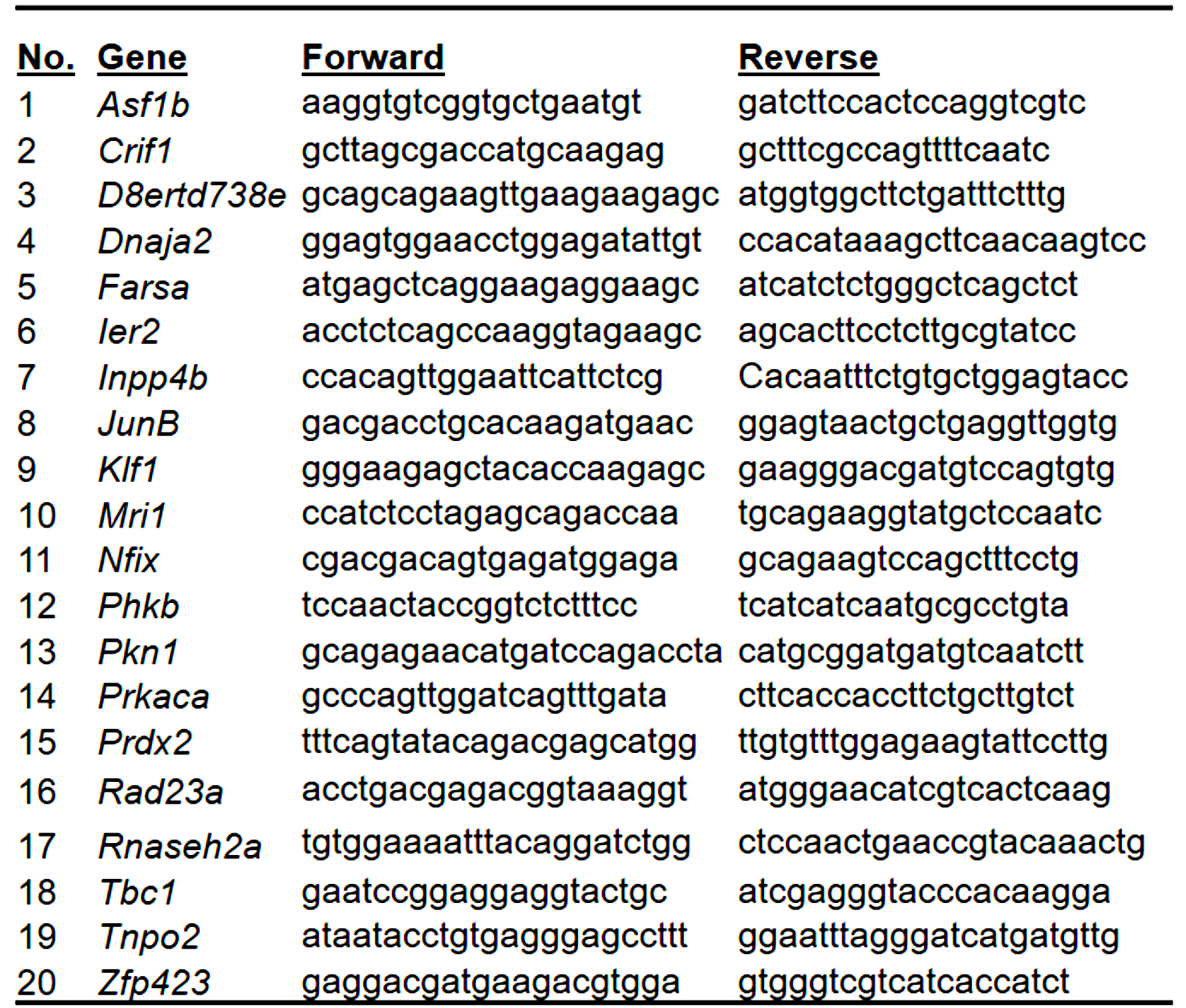

Supplement: S4 Table — (TIF) [file pone.0179033.s007.tif]

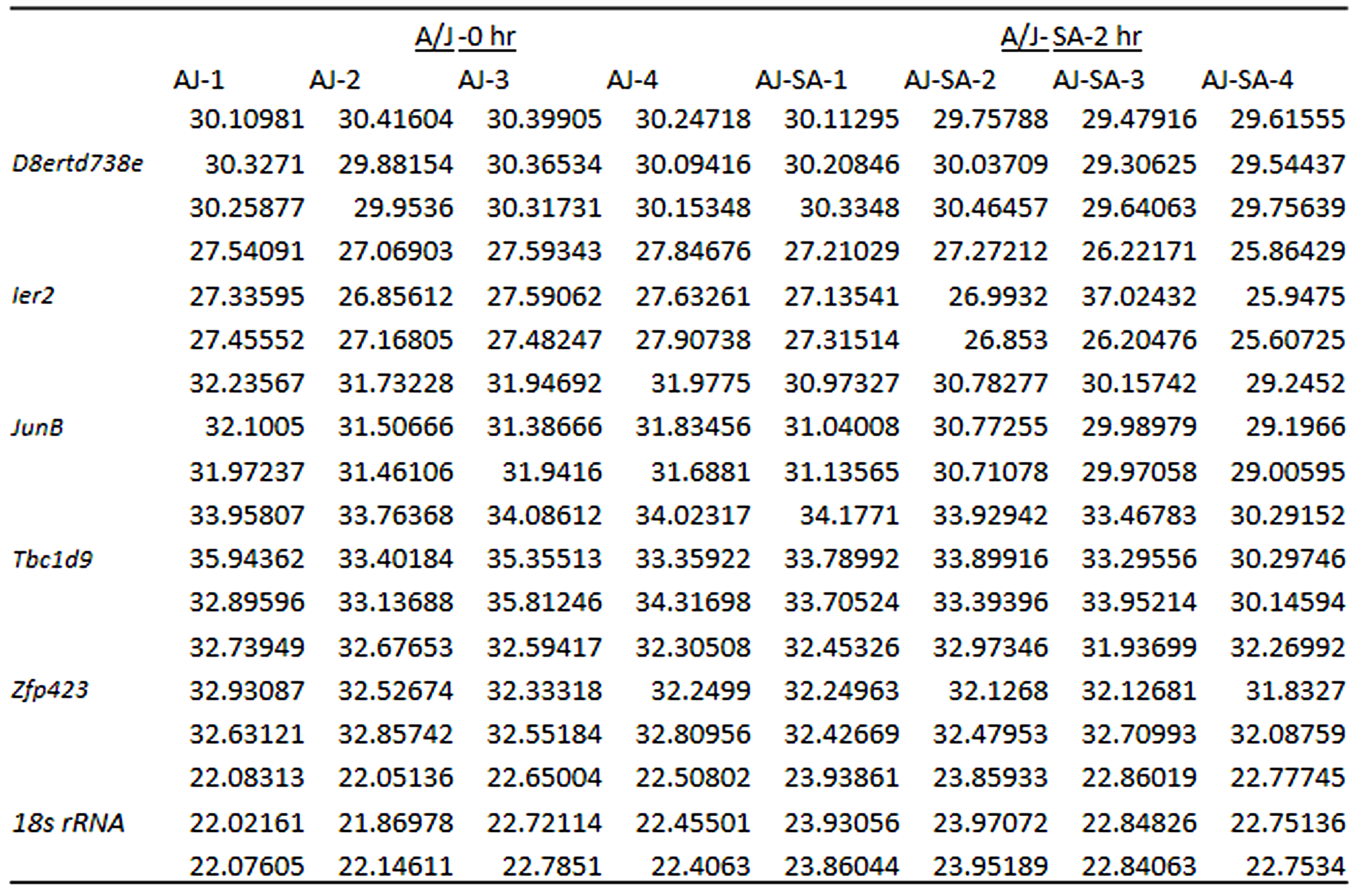

Supplement: S5 Table — A. Ct values for qPCR results of 5 candidate genes identified by Strategy 1. Male 8 week-old mice were used (n = 4 in each group). B. 18s rRNA normalized Ct values for qPCR results of 5 candidate genes identified by Strategy 1. Male 8 week-old mice were used (n = 4 in each group). (TIFF) [file pone.0179033.s008.tiff]

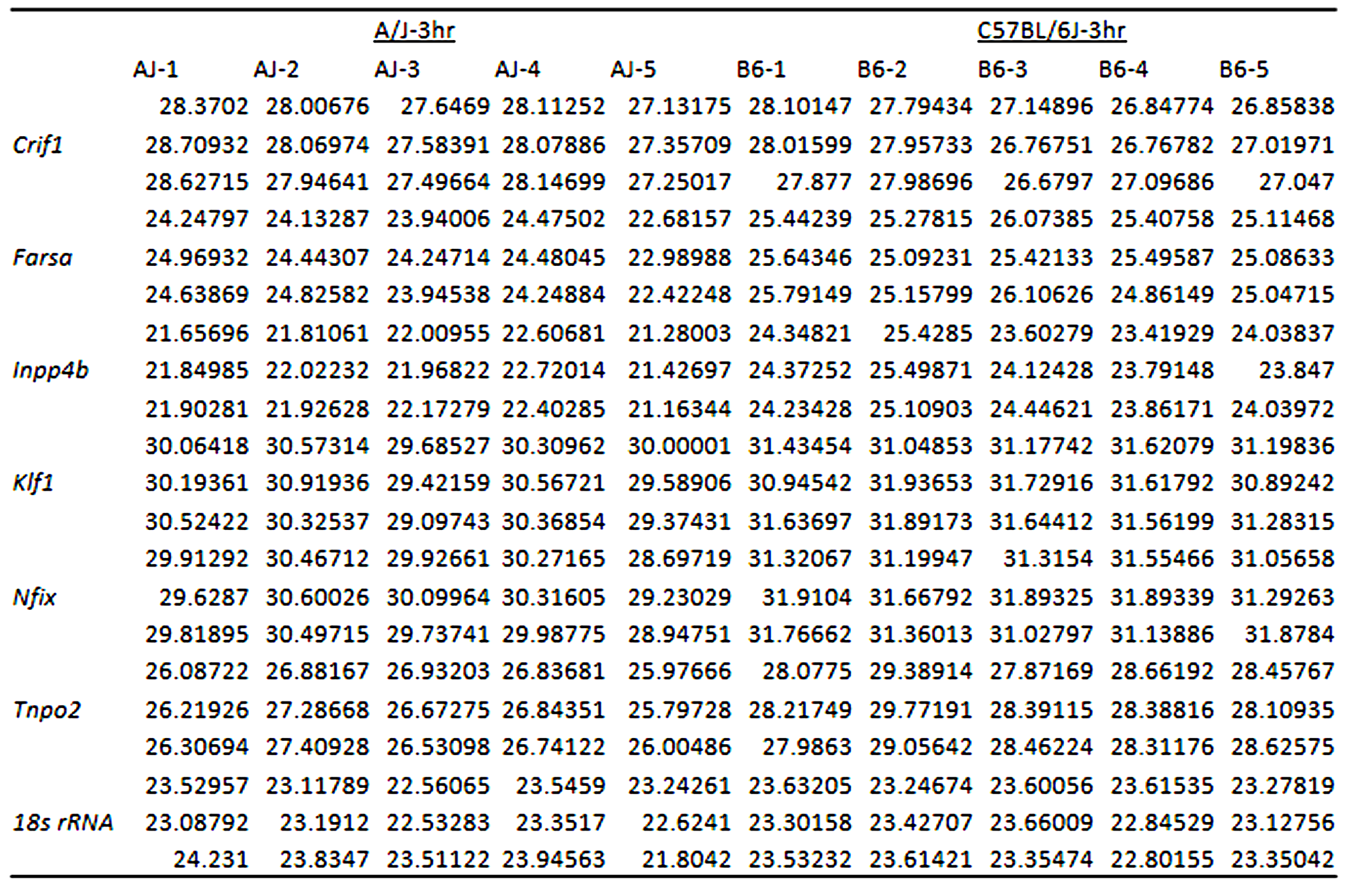

Supplement: S6 Table — A. Ct values for qPCR results of 6 candidate genes identified by Strategy 2. Male 8 week-old mice were used (n = 5 in each group). B. 18s rRNA normalized Ct values for qPCR results of 6 candidate genes identified by Strategy 2. Male 8 week-old mice were used (n = 5 in each group). (TIFF) [file pone.0179033.s009.tiff]

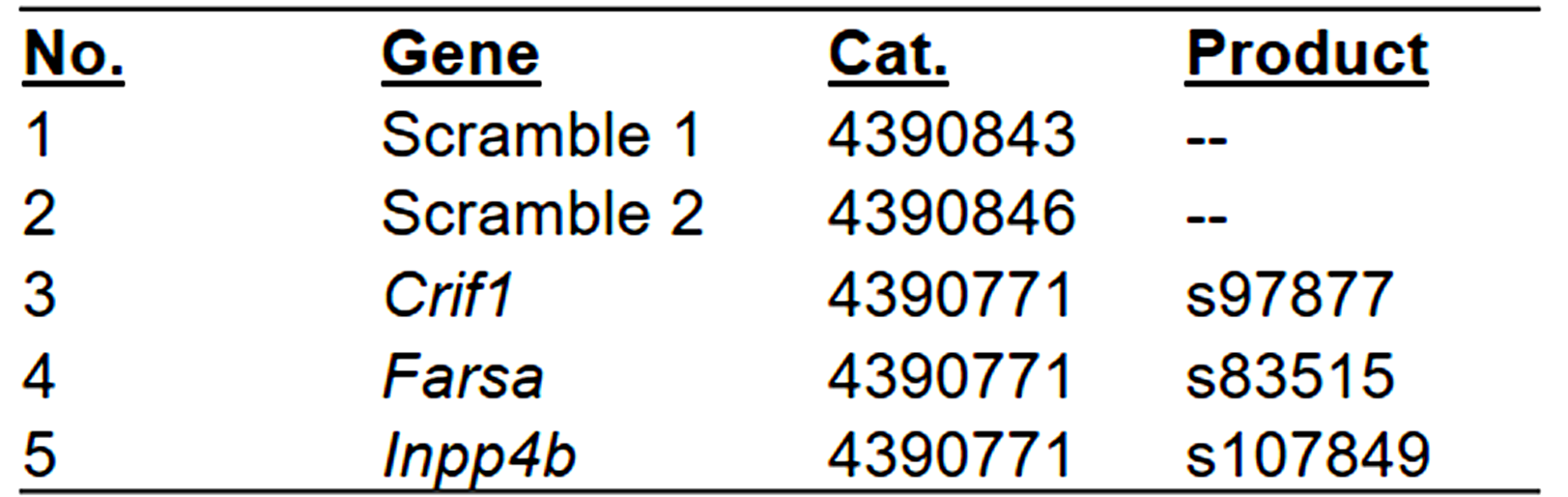

Supplement: S7 Table — (TIF) [file pone.0179033.s010.tif]
